# Japanese value set for the Functional Assessment of Cancer Therapy Eight Dimension (FACT-8D) cancer-specific preference-based quality of life instrument

**DOI:** 10.1186/s12955-025-02442-3

**Published:** 2025-10-29

**Authors:** T. Shiroiwa, M. T. King, R. Campbell, T. Murata, K. Shimozuma, T. Fukuda, R. Norman

**Affiliations:** 1 Center for Outcomes Research and Economic Evaluation for Health (C2H), National Institute of Public Health, 2-3-6 Minami, Wako, Saitama 351-0197 Japan; 2 Faculty of Science, School of Psychology, University of Sydney, Sydney, NSW Australia; 3 Crecon Medical Assessment Inc., Tokyo, Japan; 4 Comprehensive Unit for Health Economic Evidence Review and Decision Support (CHEERS), Ritsumeikan University, Kyoto, Japan; 5 School of Population Health, Curtin University, Perth, WA Australia

**Keywords:** Multi-attribute utility, Preference-based measure, Cancer, Discrete choice experiment, Functional Assessment of Cancer Therapy (FACT)-G, FACT-8D

## Abstract

**Purpose:**

The Functional Assessment of Cancer Therapy General (FACT-G) questionnaire is frequently used to assess health-related quality of life (HRQOL) in cancer patients. However, data obtained using the FACT-G cannot be directly used to calculate quality-adjusted life years (QALYs). The newly developed FACT Eight Dimensions (FACT-8D) is a preference-based measure that generates health utilities scores from 9 of the 27 FACT-G items, representing eight HRQOL domains (Nausea, Pain, Fatigue, Sleep, Work, Worry, Sadness, Support from family/friends). This study aimed to create a Japanese FACT-8D value set.

**Methods:**

A cross-sectional online survey of the Japanese general population recruited participants via a Japanese online panel, quota sampled by age (≥ 18 years) and sex. FACT-8D valuation data were collected with a discrete choice experiment. The valuation task required each participant to consider 16 pairs of hypothetical health states, randomly assigned per participant from 800 choice-sets. Preference weights were obtained from conditional logit models by dividing estimated HRQOL coefficients by the life duration coefficient.

**Results:**

Data from 2320 participants were used to assess sample representativeness and estimate the Japanese value set. All preference weights other than Worry Level 2 were negative and increased in absolute terms in progressively higher levels of adverse HRQOL impact. The most influential domains for health utilities were Pain and Nausea, followed by Work problems. Fatigue, Sleep, Support, Sadness, and health Worry had moderate influences on health utilities. The lowest score, for the pit state [55555555], was − 0.60. This value is much lower than that of the EORTC QLU-C10D pit state [4444444444], -0.22. Health states were consistently scored higher in the USA, Australia and the UK than in Japan. Canadian health states are generally lower than for Japan, but not universally so.

**Conclusions:**

We established the Japanese FACT-8D value set based on the internationally common protocol. The value set provides another option for quantifying health utilities for cancer outcomes. This contributes to improving the feasibility of deriving health utilities from the widely used FACT-G.

**Supplementary Information:**

The online version contains supplementary material available at 10.1186/s12955-025-02442-3.

## Introduction

In Japan, since 2019, economic evaluation submissions have been required for drug and medical device pricing before the Ministry of Health, Labour and Welfare can approve higher prices than those for existing drugs [1]. The new guideline for submission to the Japanese Center for Outcomes Research and Economic Evaluation for Health (C2H), revised in 2024 [2], recommends the use of quality-adjusted life years (QALYs) for outcome measurement. The quality adjustment metric used to calculate QALYs is health utility ([3]).

Each health state in an economic model requires an estimate of health utility, often called the health state utility value (HSUV). Preference-based measures (PBM) are measurement systems that provide HSUVs by enabling patients to describe the impact of ill health and assigning a utility score to those descriptions on the basis of peoples’ preferences for health states [4]. PBMs consist of two components: (1) a standardized descriptive system composed of a number of multi-level dimensions that together describe a finite set of health states; and (2) a utility algorithm of preference weights that assigns a HSUV to each health state. Together, these HSUVs constitute a value set. The most commonly recommended PBM in health technology assessment (HTA) guidelines internationally is the generic EQ-5D-5 L is [5], although condition-specific preference-based measures (CSPBMs) are also acceptable in some jurisdictions. A CSPBM is an instrument that measures the health utility of heath states specific to a particular health condition or disease [6]. Generic PBMs may lack sensitivity to clinically important differences or responsiveness to clinically important change in certain health conditions and clinical contexts. In such cases, CSPBMs may be more sensitive to both cross-sectional differences and longitudinal changes in health states.

In collaboration with the European Organisation for Research and Treatment of Cancer (EORTC) Quality of Life Group, the Multi-Attribute Utility in Cancer (MAUCa) Consortium developed EORTC QLU-C10D [7], a CSPBM for cancer. The QLU-C10D enables HSUVs to be calculated from HRQOL data collected with the EORTC QLQ-C30 [8], a self-report questionnaire widely used in clinical trials [9]. QLU-C10D value sets were then created for Japan [10] and other countries [11–21]. The MAUCa Consortium used similar methods to develop another CSPBM, the FACT-8D [22], which uses 9 of the 27 items from the Functional Assessment of Cancer Therapy-General (FACT-G) [23]. Like the EORTC QLQ-C30, the FACT-G is designed to measure HRQOL in patients with cancer and is frequently used in many countries, including Japan. Many clinical studies have used FACT-G to measure and evaluate the health state of cancer patients [9], and have accumulated significant amounts of FACT-G data. However, these data cannot be used to calculate QALYs, because the FACT-G is not preference-based. This presents a challenge for researchers in HTA and quality-of-life research.

The FACT-8D enables calculation of HSUVs from nine of the 27 FACT-G items, representing 8 HRQOL domains: Nausea, Pain, Fatigue, Sleep, Work, Worry, Sadness, Support from family/friends. Patients’ FACT-G responses are converted into FACT-8D health states and HSUVs can be calculated using a country-specific utility algorithm to yield country-specific value sets. This has been done for four countries to date: Australia [22], Canada [24], the United States (US) [25], and the United Kingdom (UK) [26]. This study aimed to produce a Japanese FACT-8D value set by applying the MAUCa Consortium’s standardised international valuation protocol in the Japanese general population, and to compare the FACT-8D Japanese value set to those from other countries.

## Methods

A cross-sectional population-based survey was designed to collect FACT-8D valuation data from a representative sample of the Japanese general population, following the FACT-8D valuation methodology designed by the MAUCa Consortium [22]. The study protocol was approved by the Japanese National Institute of Public Health ethics committee (approval number NIPH-IBRA #12272).

### Survey sampling and implementation

The survey, conducted from 21 December 2021 to 21 January 2022, collected sociodemographic and health status data. The valuation component utilized a discrete choice experiment (DCE). SurveyEngine, a company specializing in online choice experiments, managed sample recruitment (via a Japanese online panel), survey administration and data collection. SurveyEngine and its panel provider complied with the International Code on Market, Opinion and Social Research and Data Analytics [27]. Members of the online panel were eligible if 18 years or older and able to read Japanese. Online panelists received an email invitation, including a hyperlink to the survey. Any who attempted to enter the survey via mobile phones were excluded as the DCE was too complex for a small screen. Consent was sought from those who successfully entered the survey. Consenters were screened for quota sampling by age and sex to achieve representativeness of the Japanese general population for these key demographics. A target sample size of 2000 respondents was determined to provide acceptable precision for model parameter estimates based on the number of health state comparisons [28].

### Discrete choice experiment

Paired comparisons in the DCE task were used to generate preference data to estimate the preference weights of the Japanese FACT-8D utility algorithm, hence to calculate HSUVs and the Japanese value set. Health states shown in the DCE task contained nine attributes: survival duration and the FACT-8D dimensions (pain, fatigue, nausea, sleep, work, support, sadness, worry). Supplementary Table A (Online Resource 1) shows how the HRQOL attributes and levels in the DCE mapped to the FACT-8D descriptive system and FACT-G source items. In the FACT-8D descriptive system, each HRQOL dimension has five levels, corresponding to the five response options in the FACT-G source items; the FACT-8D descriptive system therefore describes over 390,000 possible health states (5^8^=390,625). Survival duration had four levels: 1, 2, 5 and 10 years.

The DCE experimental design comprised 800 choice-sets that optimized statistical efficiency in estimating the utility model parameters assuming a null prior (i.e. zero prior). Supplementary Appendix A (Online Resource 2) explains how these choice sets were constructed. Each choice-set comprised two FACT-8D health states, each described by nine attributes (eight HRQOL dimensions and duration). We simplified the cognitive task by constraining the number of attributes that differed between health states in any choice-set to five, in line with a typical number of attributes shown in the DCE tasks used to develop preference-based measure value sets [29].

The valuation task required participants to consider 16 pairs of hypothetical health states (i.e., 16 choice-sets), described as ‘Option A’ and ‘Option B’ and for each choice-set, select the health state they would prefer to live in until death. Dimensions that differed between Options A and B were highlighted in yellow, a presentation format preferred by participants in our previous DCE valuation methods experiment [30]. Supplementary Figure A shows how the choice task was explained, and Supplementary Figure B shows an example choice as seen by Japanese survey participants (Online Resource 3).

There were two levels of randomization in the DCE component of the survey: (1) each respondent was allocated to a block of 16 choice-sets from the 800 in the DCE design; and (2) which option was seen as Option A or Option B was randomized within each choice-set. The nine attributes were always presented in the same order, as previous work showed that order does not systematically bias preference weights [31].

### Other survey content

**Fig. 1:**
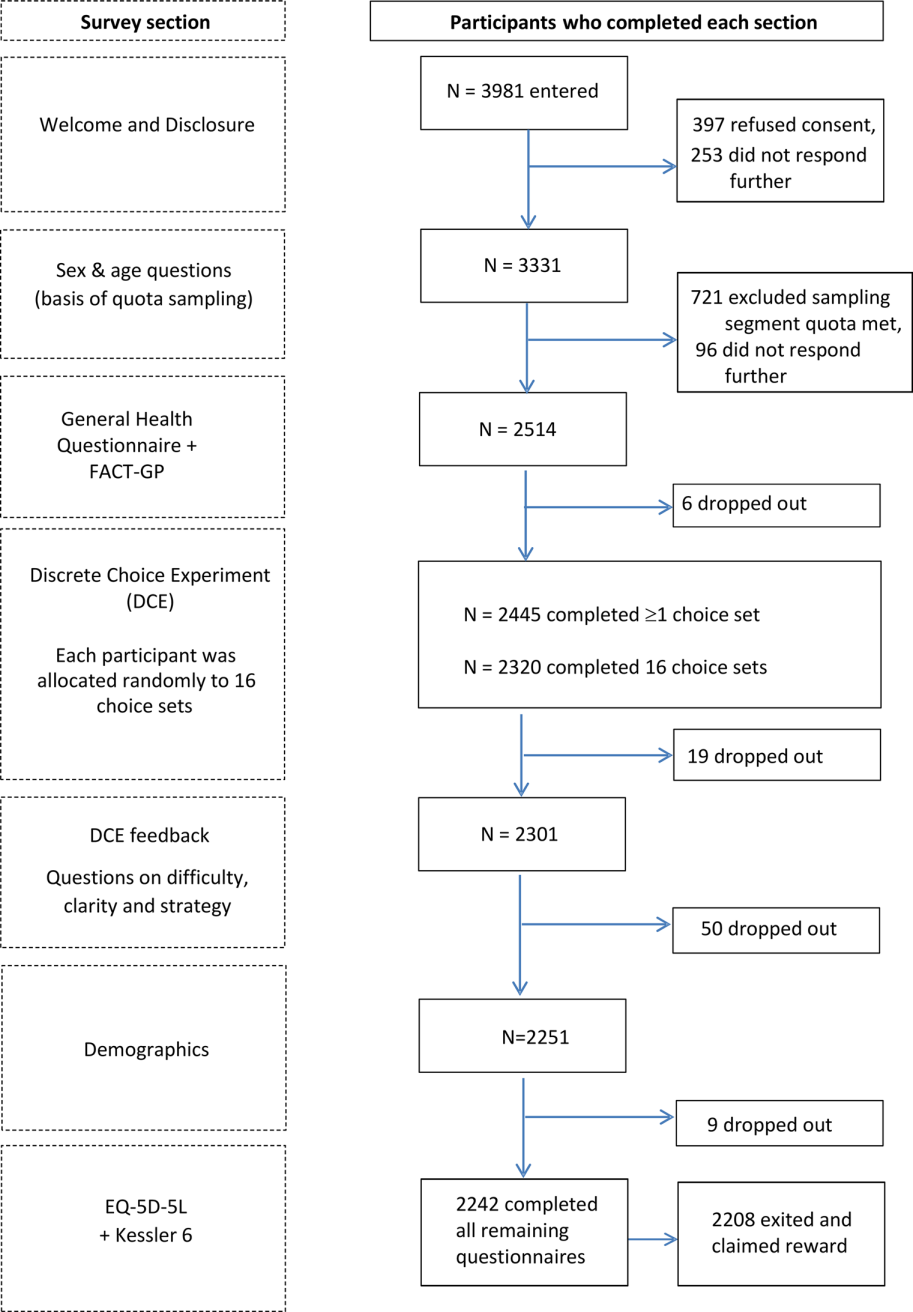
Respondent flow and sample sizes for each component of the valuation survey

The survey included sociodemographic characteristics and self-reported health measures. The order of survey components is shown in Fig. 1. After completing the DCE component, participants were asked four fixed-format questions about the difficulty and clarity of the valuation task and strategy used to choose between health states (Supplementary Appendix B, Online Resource 4).

### Statistical analyses

Descriptive statistics summarized sample demographics, self-reported general health and participant feedback on the DCE valuation task. Sample representativeness was assessed against Japanese population reference data for demographics and self-reported health using chi-square tests and t-tests.

DCE data quality was assessed by tallying how many respondents chose either all A’s or all B’s across the choice-sets and by considering the time respondents took to complete the survey. We divided respondents into deciles based on total survey time, ran a conditional logit on the DCE data in each decile, then graphed the Pseudo-R^2^ and the number of statistically significant coefficients for each decile, interpreting low values on either indicator as suggesting relatively low quality data. We also plotted the mean and distribution of time (5th, 25th, 50th, 75th and 95th percentiles) that respondents spent on each of the 16 choice sets.

The DCE data were analyzed with STATA 18.0 with two approaches: a conditional logit model and a latent class model. The conditional logit approach estimates a functional form used previously to estimate health utility from DCE data consistent with standard QALY model restrictions [31–35]. The QALY model requires a health utility scale where dead anchors the scale at zero, i.e., ‘the zero condition’ [36]. A functional form that satisfied this requirement included the interaction between the FACT-8D levels and the *TIME* variable (Eqs. 1 and 2). Therefore, as *TIME* tended to zero, the systematic component of the utility function tended to zero. Another typical requirement of the QALY model is constant proportional time trade-off [36], therefore a linear relationship between utility and *TIME* (life years) was assumed.

A useful feature of this functional form is that the impact of deviating from Level 1 (no problems) in each dimension was characterized through a two-factor interaction term with duration (the experimental design allowed for these interactions). This enabled a preference-weighting algorithm in which the effect of each level of each dimension could be included as a decrement from full health.

The analysis (Eq. 1) used conditional logit models in which the utility of option *j* in choice-set *s* for survey respondent *i* was assumed to be:

1 $$\:{U}_{isj}=\alpha\:{TIME}_{isj}+\:\beta\:{X}_{isj}^{{\prime\:}}{TIME}_{isj}+{\epsilon\:}_{isj}$$

*i* = 1, …, *I* respondents; *j* = health state options A, B; *s* = 1, …, 800 choice-sets.

where *α* is the utility associated with a life year,$\:\:{X}_{isj}^{{\prime\:}}$ is a vector of dummy variables representing the levels of the FACT-8D health state presented in option *j*, and *β* is the corresponding vector of preference weights associated with each level in each dimension within $\:{X}_{isj}^{{\prime\:}}$, for each life year. The error term $\:{\epsilon\:}_{\:isj}$ was assumed to have a Gumbel distribution. To adjust the standard errors to allow for intra-individual correlation, a clustered sandwich estimator was used via STATA’s *vce* (cluster) option. To estimate preference weights for each deviation from Level 1 (no problems) in each FACT-8D dimension, we divided each of the *β* terms by *α*, and used the delta method to estimate standard errors for these ratios [37].

Two conditional logit models were estimated. Model 1 included every decrement from the best level (i.e., Level 1, no problems) in each dimension (Eq. 1). Thus, $\:{X}_{isj}^{{\prime\:}}$ contained 32 terms (8 dimensions x [5–1] levels within each). Non-monotonicity in such models typically reflects noise, with the non-monotonic parameter estimates being not statistically different from each other [38]. Model 2 followed the same general form as Eq. 1 but imposed a restriction of monotonicity across levels of each dimension by combining adjacent non-monotonic levels. Model 2 therefore included a reduced number of estimates in *β* (the vector of preference weights). The MAUCa consortium has used this approach previously for the EORTC QLU-C10D (e.g [11, 20]). and the FACT-8D([22, 24, 25]). The impact of constraining coefficients was assessed with change in the model pseudo R^2^; ideally, the imposition of monotonicity would not reduce model fit markedly.

Sociodemographic variables that deviated statistically significantly from the Japanese general population (using the latest available census data in Japan at time of analysis) were considered for weighting in DCE models using raking, i.e. iterative proportional fitting, considering the type and degree of non-representativeness. Raking was implemented using the *ipfweight* command in STATA 13.0, with observations with missing demographic data assigned a weight of one. The degree of increased uncertainty in coefficient estimates due to weighting was assessed by calculating the percentage increase in the standard errors of the unweighted versus weighted Model 1 coefficients.

The second approach used a latent class model as a sensitivity analysis to account for preference heterogeneity in the DCE data. In this approach, we first estimated a set of latent class models with differing number of classes to determine the optimal number of latent classes by examining the Bayesian information criterion (BIC) and Akaike information criterion (AIC). The optimal model was then used to derive preference weights for each deviation from Level 1 (no problems) in each FACT-8D dimension, which were estimated by each coefficient weighted by class shares.

## Results

### Sample characteristics

As Fig. 1 shows, 3981 respondents entered the survey, 2445 (61%) of whom were within sampling quotas, consented and completed at least one choice-pair, and 2320 (58%) completed all choice-pairs. The data from these 2320 participants were included in analyses to assess representativeness and estimate the Japanese value set.

The sample was representative of the Japanese general population in terms of sex and age, but had higher education levels, reported poorer health status (EQ-5D) but better mental health (Kessler 6), were more likely to do housekeeping work or work while attending school, and less likely to be married (Table 1). The following five variables were included in the weighted model: education, EQ-5D, Kessler 6, Work type and Household income. The three remaining non-representative demographics were not included for the follow reasons: Region – the Japanese population is generally homogeneous across regions; Paid employment – correlated with Household income; Relationship status – differences per category were generally small.

**Table 1:** Self-reported sociodemographic characteristics and health of the Japanese valuation survey (*n* = 2320 participants who completed the 16 discrete choice experiment choice sets) compared with the Japanese general population

Characteristics	Category		Sample, *n*	Sample, % or mean, $\:\stackrel{-}{\varvec{x}}$	Japanese population, %	Test statistic^a^	*p* value
Sex^b^	Male		1127	48.6%	48.7%	*X*^2^ = 0.01	0.906
	Female		1193	51.4%	51.3%		
Age^b^	18–29 years		299	12.9%	14.0%	*X*^2^ = 5.27	0.383
	30–39 years		306	13.2%	13.3%		
	40–49 years		377	16.3%	17.2%		
	50–59 years		363	15.6%	15.1%		
	60–69 years		363	15.6%	15.1%		
	70 years or older		612	26.4%	25.3%		
Region^b^	Hokkaido		82	3.6%	4.2%	*X*^2^ = 166.22	< 0.001
	Tohoku		89	3.9%	7.0%		
	Kanto		998	43.4%	34.2%		
	Chubu		319	13.8%	16.8%		
	Kansai		489	21.1%	17.7%		
	Chugoku		107	4.6%	5.8%		
	Shikoku		51	2.2%	3.0%		
	Kyushu		164	7.1%	11.4%		
	Missing		21	-	-		
Work type^e^	Engaged mainly in work		1093	47.7%	49.8%	*X*^2^ = 293.05	< 0.001
	Engaged in work while attending school/housekeeping		283	12.3%	9.2%		
	Not at work		51	2.2%	1.6%		
	Unemployed person		44	1.9%	1.5%		
	Attending school		34	1.5%	5.4%		
	Housekeeping		415	18.1%	12.0%		
	Other		372	16.2%	20.5%		
	Missing		28	-	-		
Paid employment^c^	Full time worker		746	54.2%	52.5%	*X*^2^ = 70.22	< 0.001
	Part-time worker		249	18.1%	22.8%		
	Temporary worker		112	8.1%	8.3%		
	Director/Self-employer		165	12.0%	12.8%		
	Other		104	7.6%	3.7%		
Household income^d^	< JPY 1million		169	6.8%	6.4%	*X*^2^ = 618.11	< 0.001
	JPY 1 million <= < JPY 2 million		155	8.7%	12.6%		
	JPY 2 million <= < JPY 3 million		198	12.0%	13.6%		
	JPY 3 million <= < JPY 4 million		275	15.8%	12.8%		
	JPY 4 million <= < JPY 5 million		362	14.1%	10.5%		
	JPY 5 million <= < JPY 7 million		322	15.8%	16.7%		
	JPY 7 million <= < JPY 10 million		361	15.5%	15.2%		
	JPY 10 million <= < JPY 15 million		355	7.8%	8.8%		
	JPY 15 million <= < JPY 20 million		179	1.8%	2.1%		
	JPY 20 million <		42	1.7%	1.2%		
	Missing		40	-	-		
Education^e^	Elementary or Junior high school		42	1.8%	14.8%	*X*^2^ = 1022.21	< 0.001
	High school		640	28.1%	39.7%		
	College		426	18.7%	20.5%		
	University or graduate		1168	51.3%	24.3%		
	Missing		44	-	-		
Relationship status^f^	Unmarried		734	32.6%	31.6%	*X*^2^ = 63.42	< 0.001
	Married		1266	56.2%	61.3%		
	Bereaved		106	4.7%	3.2%		
	Divorced		145	6.4%	3.9%		
	Missing		69	-	-		
Kessler psychological distress scale (K6)^g^	0–4		1853	82.6%	71.0%	*X*^2^ = 148.61	< 0.001
	5–9		245	10.9%	18.7%		
	10–14		101	4.5%	7.2%		
	≥ 15		43	1.9%	2.7%		
	Missing		78	-	-		
EQ-5D-5 L VAS^h^	Age		Gender				
	20–29	Male	144	$\:\stackrel{-}{x}=$73.7	$\:\mu\:$ = 82.6	t = -5.33	< 0.001
		Female	138	$\:\stackrel{-}{x}=$ 73.4	$\:\mu\:$ = 81.2	t = -5.09	< 0.001
	30–39	Male	158	$\:\stackrel{-}{x}=$ 71.9	$\:\mu\:$ = 79.3	t = -4.75	< 0.001
		Female	145	$\:\stackrel{-}{x}=$ 72.7	$\:\mu\:$ = 79.4	t =-4.09	< 0.001
	40–49	Male	201	$\:\stackrel{-}{x}=$ 69.7	$\:\mu\:$ = 78.8	t =-6.43	< 0.001
		Female	170	$\:\stackrel{-}{x}=$ 70.9	$\:\mu\:$ = 80.1	t = -5.42	< 0.001
	50–59	Male	171	$\:\stackrel{-}{x}=$ 70.5	$\:\mu\:$ = 77.0	t = -4.56	< 0.001
		Female	184	$\:\stackrel{-}{x}=$ 76.1	$\:\mu\:$ = 79.2	t = -2.27	0.025
	60–69	Male	172	$\:\stackrel{-}{x}=$ 71.7	$\:\mu\:$ = 77.3	t = -3.83	< 0.001
		Female	173	$\:\stackrel{-}{x}=$ 76.3	$\:\mu\:$ = 80.5	t = -2.98	0.003
	70–79	Male	217	$\:\stackrel{-}{x}=$ 77.9	$\:\mu\:$ = 74.9	t = 2.96	0.003
		Female	301	$\:\stackrel{-}{x}=$ 78.8	$\:\mu\:$ = 76.9	t = 2.23	0.026
	80–89	Male	24	$\:\stackrel{-}{x}=$ 72.5	$\:\mu\:$ = 70.3	t = 0.52	0.609
		Female	34	$\:\stackrel{-}{x}=$ 72.1	$\:\mu\:$ = 68.1	t = 1.20	0.238
	Missing		88	-	-		
General Health Question^i^	Excellent [5]		110	4.7%	-		
	Very good [4]		429	18.5%	-		
	Good [3]		754	32.5%	-		
	Fair [2]		783	33.8%	-		
	Poor [1]		244	10.5%	-		
Total sample			**2320**				

a. For categorical variables, the Chi-Squared Goodness-of-Fit test was used to compare observed category frequencies with those expected based on population proportions. For continuous variables, t-test was used to compare sample means to population means

b. 2019 Population statistics data from https://www.e-stat.go.jp/stat-search/files?page=1&layout=datalist&toukei=00200524&tstat=000000090001&cycle=7&year=20190&month=0&tclass1=000001011679&result_back=1&tclass2val=0

c. Survey data (provided by *n* = 1602 survey participants who endorsed one of these four categories) were compared to 2019 Population statistics data after adjusting the general population percentages to be proportional to the four categories for which we had data. The remainder were either not in paid employment or possibly missed this question. 2019 Labour force survey data from https://www.e-stat.go.jp/stat-search/files?page=1&layout=datalist&toukei=00200531&tstat=000000110001&cycle=7&year=20190&month=0&tclass1=000001040276&tclass2=000001040283&tclass3=000001040284&result_back=1&tclass4val=0

d. 2018 Japanese “Comprehensive Survey of Living Conditions” data from https://www.e-stat.go.jp/stat-search/files?page=1&layout=datalist&toukei=00450061&tstat=000001129675&cycle=7&tclass1=000001130605&tclass2val=0

e. 2017 Japanese employment status survey data from https://www.e-stat.go.jp/stat-search/files?page=1&layout=datalist&toukei=00200532&tstat=000001107875&cycle=0&tclass1=000001107876&tclass2=000001107877&tclass3val=0

f. 2015 Japanese national census data from https://www.e-stat.go.jp/stat-search/files?page=1&layout=datalist&toukei=00200521&tstat=000001080615&cycle=0&tclass1=000001089055&tclass2=000001089056&tclass3val=0

g. 2019 Japanese “Comprehensive Survey of Living Conditions” data from https://www.e-stat.go.jp/stat-search/files?page=1&toukei=00450061&tstat=000001141126. Low scores indicate low non-specific psychological distress

h. Japanese Population Norms of EQ-5D-5 L Visual Analogue Score (VAS) (Shiroiwa, Noto and Fukida, 2021)

i. No normative data available

### Respondents’ perception of the DCE valuation task

Respondent perceptions of the DCE valuation task are provided in Online Resource 4. In summary, 25% found the health state presentation ‘clear’ or ‘very clear’ while 40% rated it as ‘unclear’ or ‘very unclear’. Regarding the choice task, only 7% found it ‘easy’ or ‘very easy’, with 67% rating it ‘difficult’ or ‘very difficult’ to choose between pairs of health states. Regarding strategy used to choose between pairs of health states, 44% focused on aspects highlighted in yellow, 24% considered most aspects, and 18% focused on just a few aspects. Additional detail on strategy provided by 209 respondents revealed that 58 (29%) focused on length of survival time. Symptoms of pain and problems with appetite and sleep were also commonly cited, with a total of 82/209 (39%) citing one or more of these symptoms.

### Data quality metrics

Supplementary Appendix C (Online Resource 5) details the data quality findings. In total, 78 of the 2,320 people who completed all choice sets answered the same for every DCE task (i.e. 3.4% gave either all A’s or all B’s across their completed choice sets). Exclusion of these 78 respondents from the unweighted constrained conditional logit made little difference (max absolute difference of 0.0027) and no evidence of bias (mean difference of -0.0015).

The median time to complete the survey was 14 minutes 29 seconds (14’29”, interquartile range 9’09” to 17’53”). Respondents in all completion time deciles sped up as they became more familiar with the choice task (Supplementary Figure C). The plots of model fit and number of statistically significant coefficients by completion time decile showed that the fastest 20% of completers had the poorest model fit and least number of statistically significant coefficients (8/33), with the greatest number of statistically significant coefficients in the middle deciles (Supplementary Figure D). Exclusion of these respondents led to relatively modest differences in the final value set; given the threshold below which exclusion might occur is arbitrary we decided to retain all data in the final analysis.

### Preference modelling

#### DCE results from conditional logit models

**Fig. 2:**
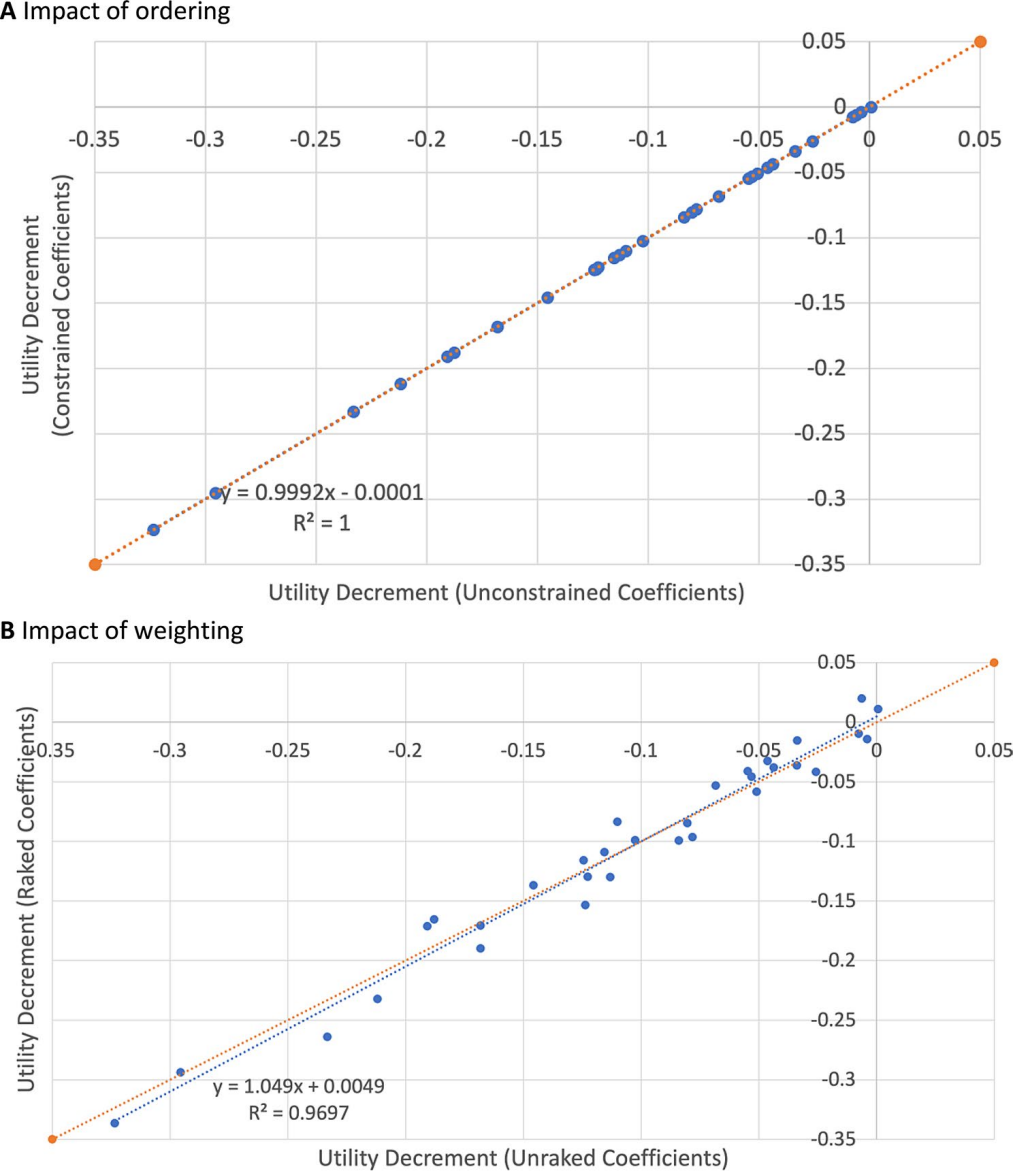
Impact of imposing monotonicity (ordering) and weighting: scatterplots of preference weights from conditional logit models

Conditional logit results for the 2320 respondents who completed all 16 choice pairs are presented in Table 2. In the unweighted Model 1 analysis, all coefficients other than Worry Level 2 were negative and increased in absolute terms in progressively higher levels. The coefficient on level 2 of the Worry dimension was very small and positive. Dimensions with the largest impact (based on the largest absolute coefficient) were Pain, Nausea and Work. The effect of constraining the coefficient on Worry level 2 was small as expected given the small positive coefficient in the unconstrained model, negligible. When responses were weighted, some small non-monotonicities were observed in the fatigue and worry dimensions. However again, the effect of combining levels to prevent this was small. Figure 2 shows the impact of enforcing monotonic ordering on the coefficients (Panel A) and using weights (Panel B). Both figures report a line of best fit between models with and without these adjustments, as well as a 45-degree line representing equality. All data points are close to the 45-degree line, illustrating minimal impact of these adjustments, and thus that the preference weights are robust to them. The standard errors of Model 1 coefficients in weighted analyses were on average 65% larger (minimum 35%, median 64%, maximum 115%).

**Table 2:** Conditional logit results for model 1 (unconstrained, unweighted) and model 2 (monotonicity imposed, unweighted and weighted) (estimated coefficients and robust standard errors (SE)), based on data from respondents who completed all 16 choice pairs in the discrete choice experiment (*n* = 2320)

Coefficient^a^ (SE)		Unweighted Analysis		Weighted Analysis^b^		Uncertainty inflation % (unconstrained)^c^
Dimension	Level	Unconstrained	Constrained	Unconstrained	Constrained	
Duration	Linear (years)	0.4343 (0.0144)***	0.4344 (0.0140)***	0.4243 (0.0257)***	0.4310 (0.0252)***	78.5
Duration x Pain	2	-0.0231 (0.0060)***	-0.0231 (0.0060)***	-0.0194 (0.0084)**	-0.0195 (0.0084)**	40.0
	3	-0.0340 (0.0064)***	-0.0340 (0.0064)***	-0.0410 (0.0101)***	-0.0409 (0.0101)***	57.8
	4	-0.0920 (0.0067)***	-0.0920 (0.0067)***	-0.0985 (0.0096)***	-0.0985 (0.0096)***	43.3
	5	-0.1405 (0.0068)***	-0.1405 (0.0068)***	-0.1428 (0.0092)***	-0.1426 (0.0093)***	35.3
Duration x Fatigue	2	-0.0027 (0.0059)	-0.0027 (0.0059)	0.0084 (0.0098)	0	66.1
	3	-0.0033 (0.0062)	-0.0033 (0.0062)	-0.0041 (0.0097)	-0.0086 (0.0073)	56.5
	4	-0.0445 (0.0060)***	-0.0445 (0.0060)***	-0.0421 (0.0101)***	-0.0464 (0.0079)***	68.3
	5	-0.0502 (0.0058)***	-0.0502 (0.0058)***	-0.0463 (0.0093)***	-0.0503 (0.0075)***	60.3
Duration x Nausea	2	-0.0297 (0.0047)***	-0.0297 (0.0047)***	-0.0225 (0.0067)***	-0.0225 (0.0067)***	42.6
	3	-0.0478 (0.0053)***	-0.0478 (0.0053)***	-0.0355 (0.0093)***	-0.0351 (0.0093)***	75.5
	4	-0.0815 (0.0056)***	-0.0815 (0.0056)***	-0.0702 (0.0092)***	-0.0697 (0.0093)***	64.3
	5	-0.1283 (0.0055)***	-0.1283 (0.0055)***	-0.1247 (0.0086)***	-0.1244 (0.0087)***	56.4
Duration x Sleep	2	-0.0190 (0.0062)***	-0.0190 (0.0062)***	-0.0161 (0.0100)	-0.0164 (0.0099)*	61.3
	3	-0.0238 (0.0057)***	-0.0238 (0.0057)***	-0.0175 (0.0103)*	-0.0176 (0.0103)*	80.7
	4	-0.0540 (0.0058)***	-0.0540 (0.0058)***	-0.0492 (0.0098)***	-0.0492 (0.0098)***	69.0
	5	-0.0829 (0.0061)***	-0.0829 (0.0061)***	-0.0726 (0.0122)***	-0.0727 (0.0121)***	100.0
Duration x Work	2	-0.0147 (0.0048)***	-0.0147 (0.0048)***	-0.0154 (0.0090)*	-0.0154 (0.0090)*	87.5
	3	-0.0349 (0.0049)***	-0.0349 (0.0049)***	-0.0359 (0.0086)***	-0.0358 (0.0086)***	75.5
	4	-0.0730 (0.0052)***	-0.0730 (0.0052)***	-0.0805 (0.0081)***	-0.0804 (0.0081)***	55.8
	5	-0.1013 (0.0053)***	-0.1013 (0.0053)***	-0.1120 (0.0085)***	-0.1117 (0.0084)***	60.4
Duration x Support	2	-0.0017 (0.0055)	-0.0017 (0.0055)	-0.0061 (0.0080)	-0.0061 (0.0078)	45.5
	3	-0.0146 (0.0059)**	-0.0146 (0.0059)**	-0.0066 (0.0098)	-0.0065 (0.0098)	66.1
	4	-0.0491 (0.0060)***	-0.0491 (0.0060)***	-0.0551 (0.0108)***	-0.0551 (0.0109)***	80.0
	5	-0.0632 (0.0058)***	-0.0632 (0.0058)***	-0.0580 (0.0102)***	-0.0581 (0.0103)***	75.9
Duration x Sadness	2	-0.0201 (0.0061)***	-0.0201 (0.0061)***	-0.0139 (0.0095)	-0.0140 (0.0095)	55.7
	3	-0.0221 (0.0057)***	-0.0221 (0.0057)***	-0.0248 (0.0085)***	-0.0253 (0.0085)***	49.1
	4	-0.0533 (0.0058)***	-0.0533 (0.0058)***	-0.0551 (0.0097)***	-0.0552 (0.0097)***	67.2
	5	-0.0730 (0.0061)***	-0.0730 (0.0061)***	-0.0724 (0.0098)***	-0.0727 (0.0097)***	60.7
Duration x Worry	2	0.0003 (0.0060)	0	0.0047 (0.0129)	0	115.0
	3	-0.0112 (0.0055)**	-0.0113 (0.0048)**	-0.0177 (0.0094)*	-0.0200 (0.0079)**	70.9
	4	-0.0364 (0.0057)***	-0.0366 (0.0047)***	-0.0422 (0.0101)***	-0.0446 (0.0068)***	77.2
	5	-0.0537 (0.0060)***	-0.0538 (0.0050)***	-0.0651 (0.0097)***	-0.0676 (0.0081)***	61.7
Pseudo R2		0.0951	0.0951	0.0965	0.0964	
Log Pseudo-likelihood		-23,321	-23,321	-20,787	-20,788	
Akaike information criterion (AIC)		46,708	46,706	41,640	41,638	
Bayesian information criterion (BIC)		47,012	47,001	41,944	41,924	

a. The coefficient for each level of each QOL domain was estimated as the interaction of that level with duration. Levels combined to ensure monotonicity within each dimension are noted in italics. Statistical significance: ***0.1%; **1%; *5%

b. Analyses were weighted for five variables simultaneously using raking: education, work type, household income, health status (EQ-5D), mental health (Kessler 6)

c. Variance inflation expressed as percentage increase in Model 1 coefficient SE = (weighted SE − unweighted SE)/unweighted SE; average (Av.), minimum (Min.), median (Med.), maximum (Max.)

**Fig. 3:**
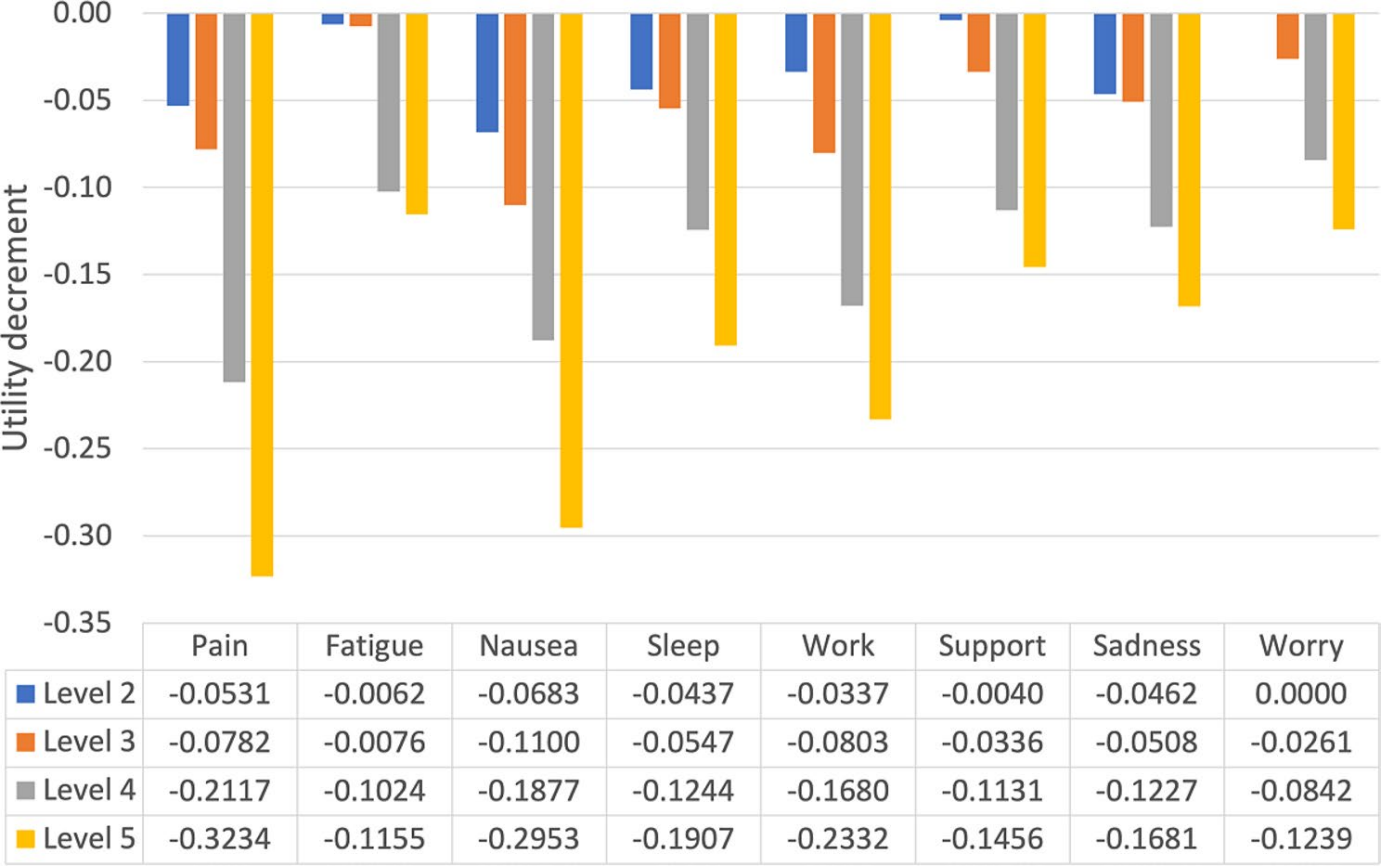
Japanese FACT-8D preference weights for each dimension and level (Model 2 conditional logit, unweighted)

The preference weights from unweighted Model 2 are plotted in Fig. 3 and tabulated under the graph. As these were derived by dividing through the coefficients in Table 2 by the duration coefficient, the pattern was unchanged, with Nausea, Pain and Work the largest drivers of preference.

As a further robustness check, the same analyses were run including all DCE data (*n* = 2445 respondents who completed at least one choice set). As shown in Supplementary Table B (Online Resource 6), results for these subsets were very similar to those in Table 2, i.e. the same to two decimal places in 29/33 cases.

#### DCE results from latent class models

Four latent class models were estimated, with two, three, four and five classes respectively. As shown in Online Resource 7,the four-class model best fit the DCE data according to AIC and BIC. The utility decrements derived from this model were compared with those derived from conditional logit Model 2 (Online Resource 8). For Levels 2 and 3, the CLOGIT utility decrements tended to be similar or smaller than those derived from the LCLogit; the exceptions were Nausea, Work, and Sadness, where the CLOGIT utility decrements were larger. For levels 4 and 5, all the CLOGIT utility decrements were larger than the corresponding LCLogit decrements across all dimensions. The pits state provides the extreme example: U(55555555) from the four-class LCLogit = -0.2074 versus CLOGIT = -0.5958, a difference of -0.3884.

#### The Japanese FACT-8D scoring algorithm

Considering all results of the various models, we recommend that the preference weights from unweighted conditional logit Model 2 be used in the Japanese FACT-8D scoring algorithm, provided in Online Resource 9, including syntax for STATA and SPSS. We recommend this model for the following reasons. Comparison of the conditional logit approach with the latent class model showed that the latter yielded systematically and substantively smaller preference weights for Levels 4 and 5 of all dimensions. In choosing the conditional logit approach, we prioritised the standardisation of statistical methodology in order to maintain unconfounded interpretation when comparing the Japanese FACT-8D scores with those of other countries. We chose the unweighted Model 2 to maintain monotonicity and because weighting did not change the coefficient estimates much but did increase standard errors considerably.

#### Japanese value set compared with other countries

**Fig. 4:**
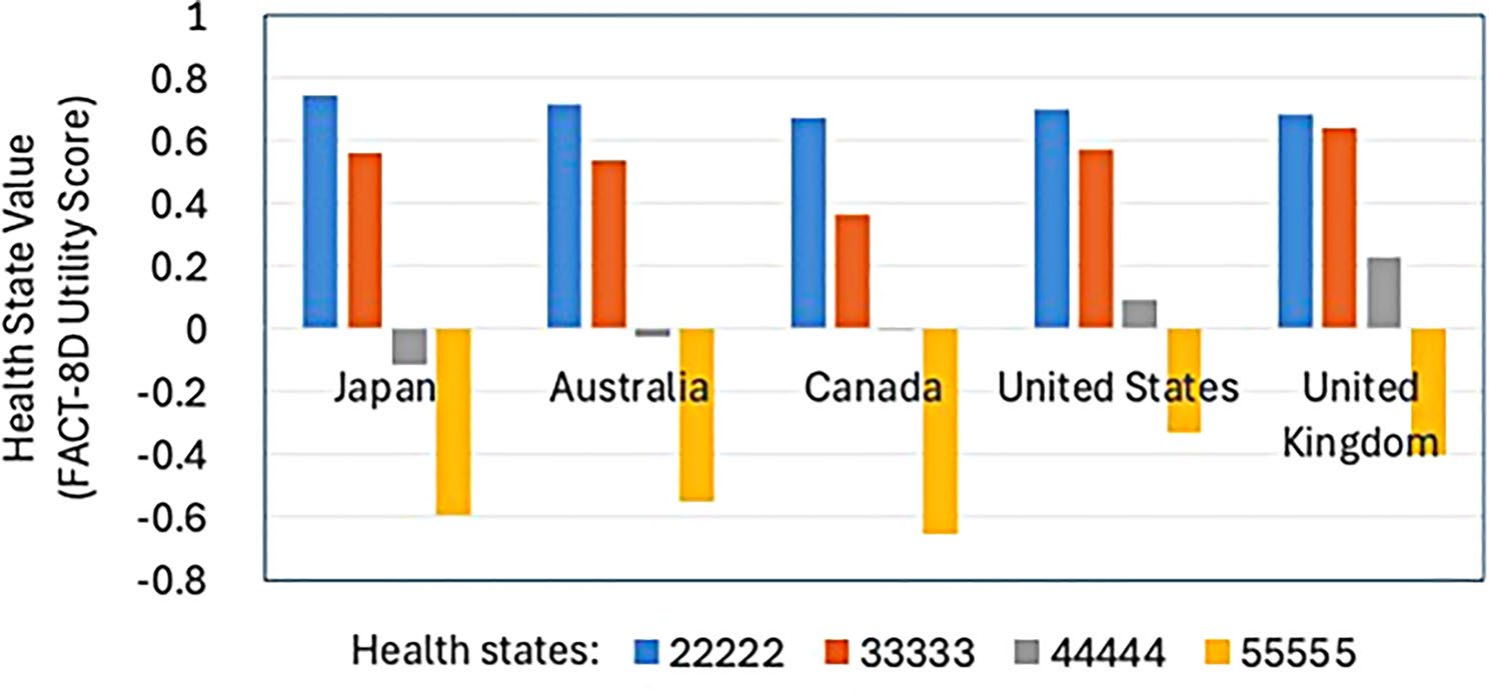
Comparison of Japanese FACT-8D utility scores for four health states with those using scoring algorithms from Australia, Canada and the United States

The first comparison was based on four health states representing a range of health from very good to worst possible, with utility scores based on 4 country-specific utility algorithms (Fig. 4). For the milder two health states (22222222 and 33333333) the Japanese value set placed the health state in a similar value to the Australian and United States value set, but somewhat higher than under the Canadian value set for 33,333,333. For the more severe health states, the Japanese values were the lowest of the four countries for 44,444,444, and second lowest for the pits state (55555555).

**Fig. 5:**
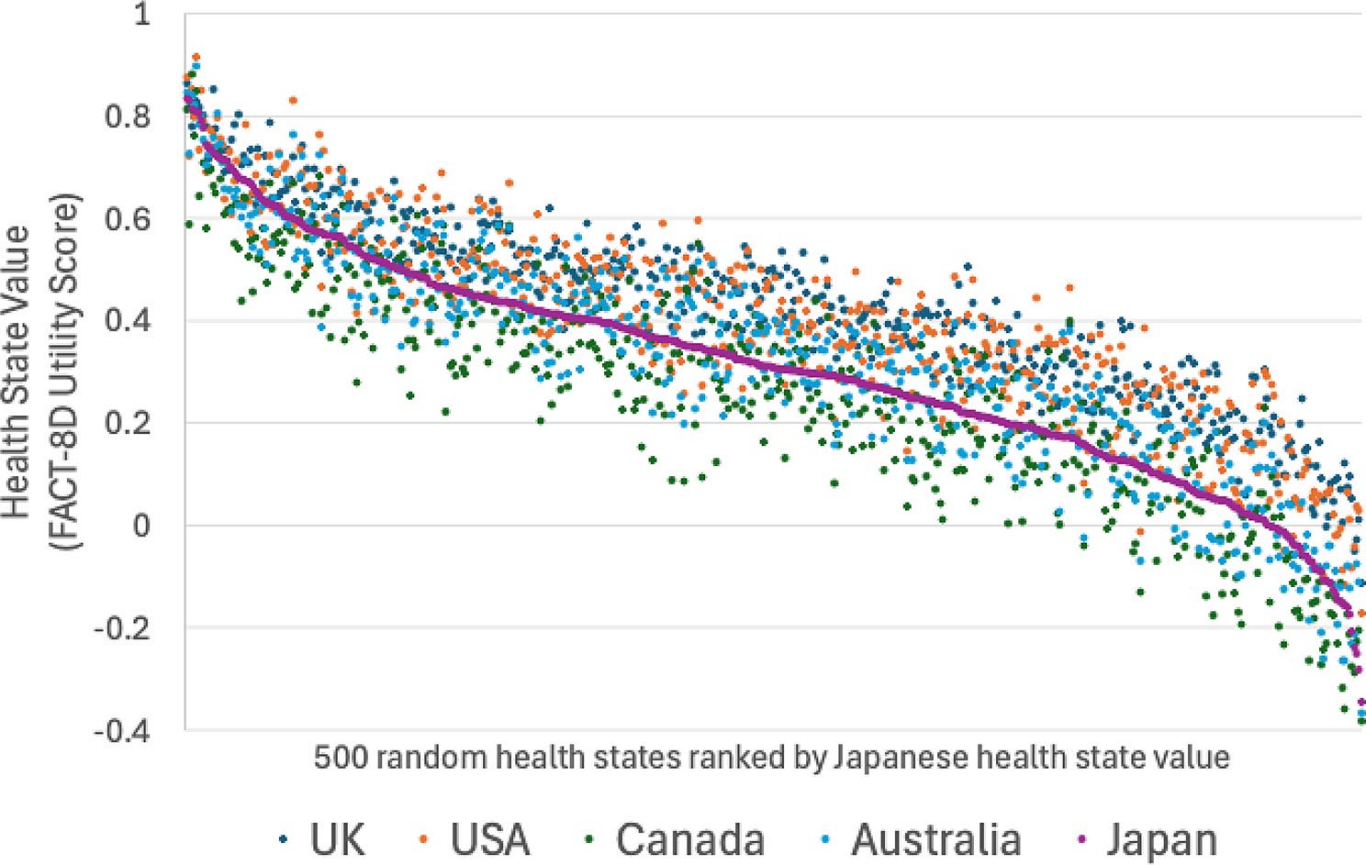
FACT-8D health state values for Japan, Australia, Canada and United States for 500 random health states

The second comparison reiterates this pattern for 500 random health states (Fig. 5). Health states were consistently scored higher in the USA and Australia than in Japan. Canadian health states were generally lower than for Japan, but not universally so.

## Discussion

This is the first report of the development of a FACT-8D value set for the Asian region. In the Japanese FACT-8D value set we developed, the most influential domains for FACT-8D HSUVs are Pain and Nausea, followed by Problems with Work. Fatigue, Sleep, Support, Sadness, and Worry have moderate influences on FACT-8D HSUVs. Similar to existing value sets in Australia, Canada, the UK and the US, Pain and Nausea are the most important impacts on utility. Conversely, the impact of sleep in Japan is higher than in other countries (Coefficients of level 5 = -0.190, compared to those in Australia, the US, Canada, and the UK which are − 0.112, -0.077, -0.075 and − 0.121, respectively). Sleep is the fourth most influential domain in Japan, but the least influential among the other four countries. As a result, the weight of level 5 for Fatigue (-0.1155) is larger than that for Sleep, but only in Japan. Comparison of preferences regarding Work and Support may also be of interest. In Japan, the worst level of the Work item is less preferred than the worst level of the Support item, although the preference for these is similar in the other countries. The difference between the worst coefficients of Work and Support is -0.0876 (-0.2332 [Work]–(-0.1456 [Support])), which is the largest among the four countries (-0.009, 0.0103, -0.0230, and − 0.010 in Australia, the US, Canada and the UK respectively). This may reflect the Japanese characteristic whereby people are hesitant to rely on others, and they try to solve their problems by themselves. As a methodological footnote, we can have confidence in our interpretation of similarities and differences in preference patterns among countries because we used the same valuation protocol and statistical modelling across all countries. Otherwise, it would not be possible to determine whether observed differences reflected true between-country differences (e.g. due to differences in underlying cultural values), or whether they were methodological artefacts induced by different valuation and statistical approaches.

In previous work, a Japanese value set was estimated for the cancer-specific EORTC QLU-C10D. In that value set [10], Physical function (PF) had the greatest impact on health utility (coefficient of the worst level is-0.2667). Pain was the most influential in the symptom items (-0.1695), although the lowest coefficient of FACT-8D is larger at -0.3224. Among the symptom items in the QLU-C10D (Pain, Fatigue, Sleep, Appetite, Nausea, and Bowel problems), the second largest decrement is observed for the worst level of Nausea (-0.1292), which is consistent with the Japanese value set of FACT-8D. In the EORTC QLU-C10D, the order of dimension importance is Pain, Nausea, Fatigue (-0.0844), Bowel problems (-0.0792), Appetite (-0.0776), and Sleep (-0.0761). In contrast to FACT-8D, the impact of fatigue is slightly greater than that of sleep. For the Japanese EORTC QLU-C10D value set, the value of the worst health state, the ‘pit state’ [4444444444], is -0.22, which is the second lowest among the existing 12 published QLU-C10D country-specific value sets, with France being the lowest (-0.44). In the case of the FACT-8D, the lowest score [55555555] is -0.60, which is much lower than that of the EORTC QLU-C10D pit state. Referring to FACT-8D value sets of other countries, the Japanese lowest score is comparable with that of Australia (-0.54), Canada (-0.65), and the US (-0.33).

The Japanese domain-specific preference weights had only one inconsistency (i.e. a better domain level but a larger preference decrement) at the first and second levels of Worry. In contrast, other countries had more inconsistencies: 8 in Australia, 9 in Canada, and 11 in the US. This suggests better data quality in the Japanese DCE data, as the observed preference weights adhered almost perfectly to the expected logical ordering of levels within domains. Factors that may have contributed to better data quality include the new experimental design we devised for the Japanese DCE, and cultural differences in respondents’ attitudes to the choice task. According to the respondents’ feedback in Supplementary Appendix B, the percentage of Japanese respondents who selected “very difficult” or “difficult” for the question (“How difficult was it to choose between the pairs of health states on each screen?”) was approximately 67%, whereas it was approximately 40% in the other four countries. Similarly, approximately 40% of Japanese respondents rated the presentation of the health states as “unclear” or “very unclear,” which is 10% less than for the other four countries. Conversely, regarding choice strategy, 44% of Japanese respondents “focused on the aspects that were highlighted in yellow” and only 7.6% “considered all of the aspects.” In other countries, the percentages were about 20–25% and 24–30%, respectively. This suggests the Japanese respondents may have engaged with the DCE choice task more seriously and modestly, and adhered to instructions more attentively than those in Australia, Canada, UK and US.

The FACT-G is frequently used, particularly in the cancer field [9]. Before the FACT-8D was created, data obtained using the FACT-G could not be used directly to calculate QALYs. Therefore, when FACT-G data was used for cost-effectiveness analysis, mapping from the FACT-G to PBMs was often used [39]. However, mapping is considered a controversial method [40]. In the Japanese HTA guidelines, mapping is only allowed if utility data cannot be obtained by other methods. Using the FACT-8D value set, we can directly obtain health utilities from FACT-G responses. We can expect to obtain less uncertain health utility scores from cancer-specific HRQOL instruments. In addition, cancer-specific instruments such as the FACT-G or EORTC QLQ-C30 may be more sensitive to clinically important differences between groups and changes over time than generic instruments including EQ-5D-5 L. A growing body of evidence partially supports this expectation for the QLU-C10D, with limited evidence to date for the FACT-8D. Finally, many clinical trials have collected FACT-G data in Japan and health utilities can be calculated from such accumulated data. Considering these points, the Japanese value set for the new tool, FACT-8D, is beneficial for academics and the Japanese HTA system.

The FACT-8D descriptive system is a critical component of this CSPBM, so warrants some discussion. Details of its development are provided in the first FACT-8D paper [22], with further detail of the same methods used by our team to develop the descriptive system of the QLU-C10D [7]. In summary, the development process was psychometrically thorough, using a large pooled data set representing a wide range of cancers and treatments, plus the inclusion of both oncologist and patient opinion, supporting the clinical validity and applicability of the descriptive system. We used a DCE approach similar to one that we had previously established as feasible [31] and reliable [41].

The current study has certain strengths and limitations. We simplified our DCE choice task by not asking respondents to trade across all dimensions at once (only 5 yellow highlighted dimensions differed between pairs of health states in a choice set). As a result, the experiment design was not orthogonal; however, it does not lead to bias in the estimated coefficients. As this study was based on an international standardised protocol developed by the MAUCa Consortium [22], we can easily interpret the comparison of results across countries. The sample size of the valuation survey was sufficiently large, with quota sampling of sex and age. This enabled us to collect a representative sample comparable to the general population on these two key parameters. However, our sample was not representative in all sociodemographics. We therefore ran weighted models, and found that the preference weights obtained using the weighted and unweighted models were similar. However, we acknowledge the possibility of confounding due to unmeasured variables that affect people’s preferences. A further potential limitation was that the survey was conducted during the COVID-19 outbreak. However, whether an impact on health state preferences occurred during the pandemic period and whether the impact continues for a long time after the end of the pandemic is unknown [42, 43]. A final consideration is the choice of statistical model. We acknowledge that preference heterogeneity is likely to exist within communities. However, for HTA purposes, existing Japanese decision-making is based on mean population preferences, which are typically well captured in conditional logit models. We therefore feel conditional logit modeling was adequate for our purposes in this paper, and facilitated valid interpretation of between-country differences, which was a key aim of this study. However, as identified across three reviews of DCE for health state valuation covering the periods 1999–2018 [29], 2007–2018 [44], and 2019–2022 [45], while conditional logit has historically been the dominant modelling approach, increasingly models that account for preference heterogeneity such as mixed logits and latent class logits are being used. The role of preference heterogeneity in deriving value sets for multi-attribute utility instruments (MAUIs) deserves further consideration and discussion by the international health economic research community. The conceptual validity of different modelling approaches for deriving value sets for MAUIs needs to be scrutinised and supplemented by empirical comparisons. There is now a wealth of existing datasets that could be reanalysed to study the impact of different modelling approaches on resultant value sets. These include numerous datasets for both EORTC QLU-C10D and FACT-8D produced by the MAUCa consortium with standardized valuation methodology such that effect of modelling can be isolated. We note that currently there are some practical barriers to implementing such models for MAUIs with a relatively large number of dimensions and levels, such as FACT-8D and EORTC QLU-C10D, including long processing times, and limits to the number of random coefficients that can be estimated using common off-the-shelf software and code.

## Conclusion

The Japanese FACT-8D value set provides another option for quantifying health utilities for cancer outcomes for Japanese HTA, and improves the feasibility of deriving health utilities from the widely used FACT-G with greater certainty than indirect methods such as mapping. Ultimately, we expect that Japanese FACT-8D value set will lead to better-informed medical resource allocation for cancer care in Japan.

## Supplementary Information

Below is the link to the electronic supplementary material.

Supplementary Material 1

Supplementary Material 2

Supplementary Material 3

Supplementary Material 4

Supplementary Material 5

Supplementary Material 6

Supplementary Material 7

Supplementary Material 8

Supplementary Material 9

## Data Availability

The datasets generated and/or analyzed during the current study are not publicly available due to the lack of consent from participants, but are available from the corresponding author upon reasonable request.

## Abbreviations

C2H: Center for Outcomes Research and Economic Evaluation for Health
CSPBM: Condition-specific preference-based measure
DCE: Discrete choice experiment
EORTC: European Organisation for Research and Treatment of Cancer
FACT: The Functional Assessment of Cancer Therapy
HTA: Health technology assessment
MAUCa: Multi-Attribute Utility in Cancer
PBM: Preference-based measures
QALY: Quality adjusted life year
