# Supplementary material for: Japanese value set for the Functional Assessment of Cancer Therapy Eight Dimension (FACT-8D) cancer-specific preference-based quality of life instrument
Source: Health Qual Life Outcomes. 2025 Oct 29;23:109. doi: 10.1186/s12955-025-02442-3 (PMC12574001; doi:10.1186/s12955-025-02442-3)
Supplement: Supplementary file 1 — Supplementary Material 1 [file 12955_2025_2442_MOESM1_ESM.docx]

**Supplementary Table A.** Mapping between the FACT-8D descriptive system (dimensions and levels), FACT-G items, and attributes and levels in the discrete choice experiment

| **FACT-8D Dimension** | **DCE Attribute wording** | **FACT-8D Level** | **Descriptor** | **FACT-G item^a^ scores^b^** |
| --- | --- | --- | --- | --- |
| Pain | Pain | 1 | Not at all | GP4 = 0 |
|  |  | 2 | A little bit | GP4 = 1 |
|  |  | 3 | Somewhat | GP4 = 2 |
|  |  | 4 | Quite a bit | GP4 = 3 |
|  |  | 5 | Very much | GP4 = 4 |
| Fatigue | Fatigue | 1 | Not at all | GP1 = 0 |
|  |  | 2 | A little bit | GP1 = 1 |
|  |  | 3 | Somewhat | GP1 = 2 |
|  |  | 4 | Quite a bit | GP1 = 3 |
|  |  | 5 | Very much | GP1 = 4 |
| Nausea | Nausea | 1 | Not at all | GP2 = 0 |
|  |  | 2 | A little bit | GP2 = 1 |
|  |  | 3 | Somewhat | GP2 = 2 |
|  |  | 4 | Quite a bit | GP2 = 3 |
|  |  | 5 | Very much | GP2 = 4 |
| Sleep^c^ | Problems sleeping | 1 | Not at all | GF5 = 4 |
|  |  | 2 | A little bit | GF5 = 3 |
|  |  | 3 | Somewhat | GF5 = 2 |
|  |  | 4 | Quite a bit | GF5 = 1 |
|  |  | 5 | Very much | GF5 = 0 |
| Work^c^ | Problems doing work including work at home | 1 | Not at all | GF1 = 4 |
|  |  | 2 | A little bit | GF1 = 3 |
|  |  | 3 | Somewhat | GF1 = 2 |
|  |  | 4 | Quite a bit | GF1 = 1 |
|  |  | 5 | Very much | GF1 = 0 |
| Support^c,d^ | Problems with support from my family and/or friends | 1 | Not at all | max(GS2, GS3) = 4 |
|  |  | 2 | A little bit | max(GS2, GS3) = 3 |
|  |  | 3 | Somewhat | max(GS2, GS3) = 2 |
|  |  | 4 | Quite a bit | max(GS2, GS3) = 1 |
|  |  | 5 | Very much | max(GS2, GS3) = 0 |
| Sadness | Sadness | 1 | Not at all | GE1 = 0 |
|  |  | 2 | A little bit | GE1 = 1 |
|  |  | 3 | Somewhat | GE1 = 2 |
|  |  | 4 | Quite a bit | GE1 = 3 |
|  |  | 5 | Very much | GE1 = 4 |
| Worry my health will get worse | Worry my health will get worse | 1 | Not at all | GE6 = 0 |
|  |  | 2 | A little bit | GE6 = 1 |
|  |  | 3 | Somewhat | GE6 = 2 |
|  |  | 4 | Quite a bit | GE6 = 3 |
|  |  | 5 | Very much | GE6 = 4 |

1. Nine FACT-G items are included in the FACT-8D: GP4 (*I have pain*), GP1 (*I have a lack of energy*), GP2 (*I have nausea*), GF5 (*I am sleeping well*), GF1 (*I am able to work including work at home*), GS2 (*I get emotional support from my family*) and GS3 (*I get support from my friends*), GE1 (*I feel sad*), GE6 (*I worry that my condition will get worse*).
2. FACT-G item scores correspond to the following response options: 0 (*Not at all*), 1 (*A little bit*), 2 (*Somewhat*), 3 (*Quite a bit*), 4 (*Very much*).
3. Because the FACT-G items that determine the FACT-8D dimensions *Sleep*, *Work*, *Support* are positively framed, reverse scoring is required so that FACT-8D Level 0 represents the best score and Level 4 represents the worst score across all dimensions .
4. The FACT-8D *Support* dimension contains two items; the FACT-8D level allocated is the maximum score of the FACT-G items GS2 and GS3, i.e. the best level of support, whether from family or friends.
