# Supplementary material for: Japanese value set for the Functional Assessment of Cancer Therapy Eight Dimension (FACT-8D) cancer-specific preference-based quality of life instrument
Source: Health Qual Life Outcomes. 2025 Oct 29;23:109. doi: 10.1186/s12955-025-02442-3 (PMC12574001; doi:10.1186/s12955-025-02442-3)
Supplement: Supplementary file 2 — Supplementary Material 2 [file 12955_2025_2442_MOESM2_ESM.docx]

**Supplementary Appendix A. Construction of the discrete choice experiment choice sets**

Written by Professor Deborah Street, Centre for Health Economics Research and Evaluation, University of Technology, Sydney, Australia

[Deborah.Street@uts.edu.au](about:blank)

For any generator-developed design an initial design and a set of generators are required. In this case, since the instrument has 8 dimensions each with 5 levels, we started with the first 8 columns of the 50 run orthogonal array given at http://support.sas.com/techsup/technote/ts723_Designs.txt. To reduce the number of dominated pairs in the final design, we added 1 mod 5 to the entries in column 2, 2 mod 5 to the entries in column 4, 3 mod 5 to the entries in column 6 and 4 mod 5 to the entries in column 8. This meant that neither of the health states 11111111 or 55555555 appeared in the initial design. As we wanted to be able to estimate an interaction between duration and each of the dimensions of the instrument, and since we decided that duration should have 4 levels since that has worked well in the past, we appended each level of duration to all of the runs of the orthogonal array, giving us a total of 200 runs in the initial design used to generate the choice sets. To reduce task complexity, we decided to have only 5 of the 9 items different between the options in each choice set; that is, we decided to have the options in each choice set overlap on 4 of the dimensions. This means that every generator needs to have 4 zero entries. If we wanted to be able to ensure that every pair of attributes was overlapped together the same number of times we would need 18 generators and this was clearly impractical. Instead we elected to find a set of 4 generators, and hence 800 choice sets, in which each attribute was overlapped in 200 (two attributes) or 400 (7 attributes) of the choice sets.
