# Supplementary material for: Japanese value set for the Functional Assessment of Cancer Therapy Eight Dimension (FACT-8D) cancer-specific preference-based quality of life instrument
Source: Health Qual Life Outcomes. 2025 Oct 29;23:109. doi: 10.1186/s12955-025-02442-3 (PMC12574001; doi:10.1186/s12955-025-02442-3)

**Online Resource 3**

**Supplementary Figure A.** Explanation of the choice task from the English version of the FACT-8D valuation survey


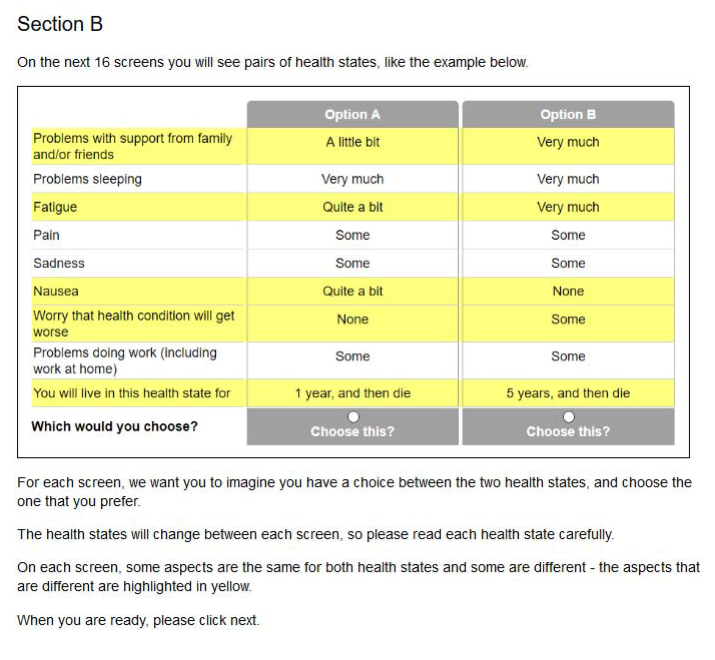


**Supplementary Figure B.** An example choice set from the discrete choice experiment valuation task as seen by Japanese survey participants


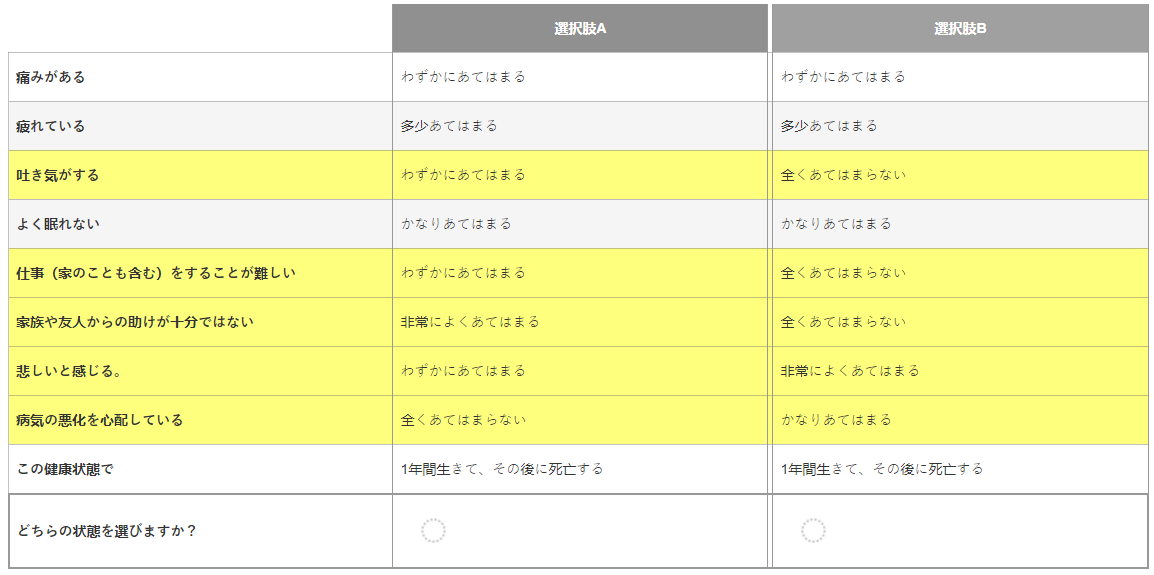

Supplement: Supplementary file 3 — Supplementary Material 3 [file 12955_2025_2442_MOESM3_ESM.docx]
