# Supplementary material for: Japanese value set for the Functional Assessment of Cancer Therapy Eight Dimension (FACT-8D) cancer-specific preference-based quality of life instrument
Source: Health Qual Life Outcomes. 2025 Oct 29;23:109. doi: 10.1186/s12955-025-02442-3 (PMC12574001; doi:10.1186/s12955-025-02442-3)
Supplement: Supplementary file 4 — Supplementary Material 4 [file 12955_2025_2442_MOESM4_ESM.docx]

**Supplementary Appendix B.** Respondent perceptions of the discrete choice valuation task

The survey included four fixed-format questions and an open-ended question to elicit respondent perceptions about the discrete choice valuation task and strategies used to choose within each choice set.

**Fixed-format questions**

*Survey question: Did you find the questions on these 16 screens easier or harder than most surveys you do?*

| Response | Frequency | Percent |
| --- | --- | --- |
| Easier | 131 | 5.7% |
| About the same | 513 | 22.3% |
| Harder | 1528 | 66.4% |
| I couldn’t say | 129 | 5.6% |
| Total | **2301** | **100.0%** |

*Survey question: How clear was the presentation of the health states?*

| Response | Frequency | Percent |
| --- | --- | --- |
| Very unclear | 143 | 6.2% |
| Unclear | 767 | 33.3% |
| Neither clear nor unclear | 816 | 35.5% |
| Clear | 520 | 22.6% |
| Very clear | 55 | 2.4% |
| Total | **2301** | **100.0%** |

*Survey question: How difficult was it to choose between the pairs of health states on each screen?*

| Response | Frequency | Percent |
| --- | --- | --- |
| Very difficult | 341 | 14.8% |
| Difficult | 1201 | 52.2% |
| Neither easy nor difficult | 597 | 25.9% |
| Easy | 131 | 5.7% |
| Very easy | 31 | 1.3% |
| Total | **2301** | **100.0%** |

*Survey question: Did you have a strategy for choosing between the pairs of health states on each screen?*

| Response | Frequency | Percent |
| --- | --- | --- |
| I did not have a strategy | 105 | 4.6% |
| I focused on just a few aspects of the health states | 424 | 18.4% |
| I focused on the aspects that were highlighted in yellow | 1022 | 44.4% |
| I considered most of the aspects | 542 | 23.6% |
| I considered all of the aspects | 176 | 7.6% |
| Other | 32 | 1.4% |
| Total | **2301** | **100.0%** |
