# Supplementary material for: Japanese value set for the Functional Assessment of Cancer Therapy Eight Dimension (FACT-8D) cancer-specific preference-based quality of life instrument
Source: Health Qual Life Outcomes. 2025 Oct 29;23:109. doi: 10.1186/s12955-025-02442-3 (PMC12574001; doi:10.1186/s12955-025-02442-3)

**Online Resource 5**

**Supplementary Appendix C.** Data Quality Results

In total, 78 of the 2,320 people who completed all choice sets answered the same for every DCE task (i.e. 3.4% gave either all A’s or all B’s across their completed choice sets). When the data from these 78 respondents were excluded and the unweighted constrained conditional logit was re-estimated, there was little difference (max absolute difference of 0.0027) and no evidence of bias (mean difference of -0.0015).

Statistics on time for survey completion were: median 14 minutes 29 seconds (14’29”), interquartile range 9’09” to 17’53”, minimum 2’48”, maximum 60’03”.

Figure C shows that respondents in all completion time deciles sped up as they became

more familiar with the choice task.

Figure D shows the fastest completion time decile yielded the least statistically significant coefficients (8/33) and those in the middle deciles yielded the most (up to 24/33 in the 4^th^ decile).

**Supplementary Figure C.** Distribution of time to completion by choice set

**
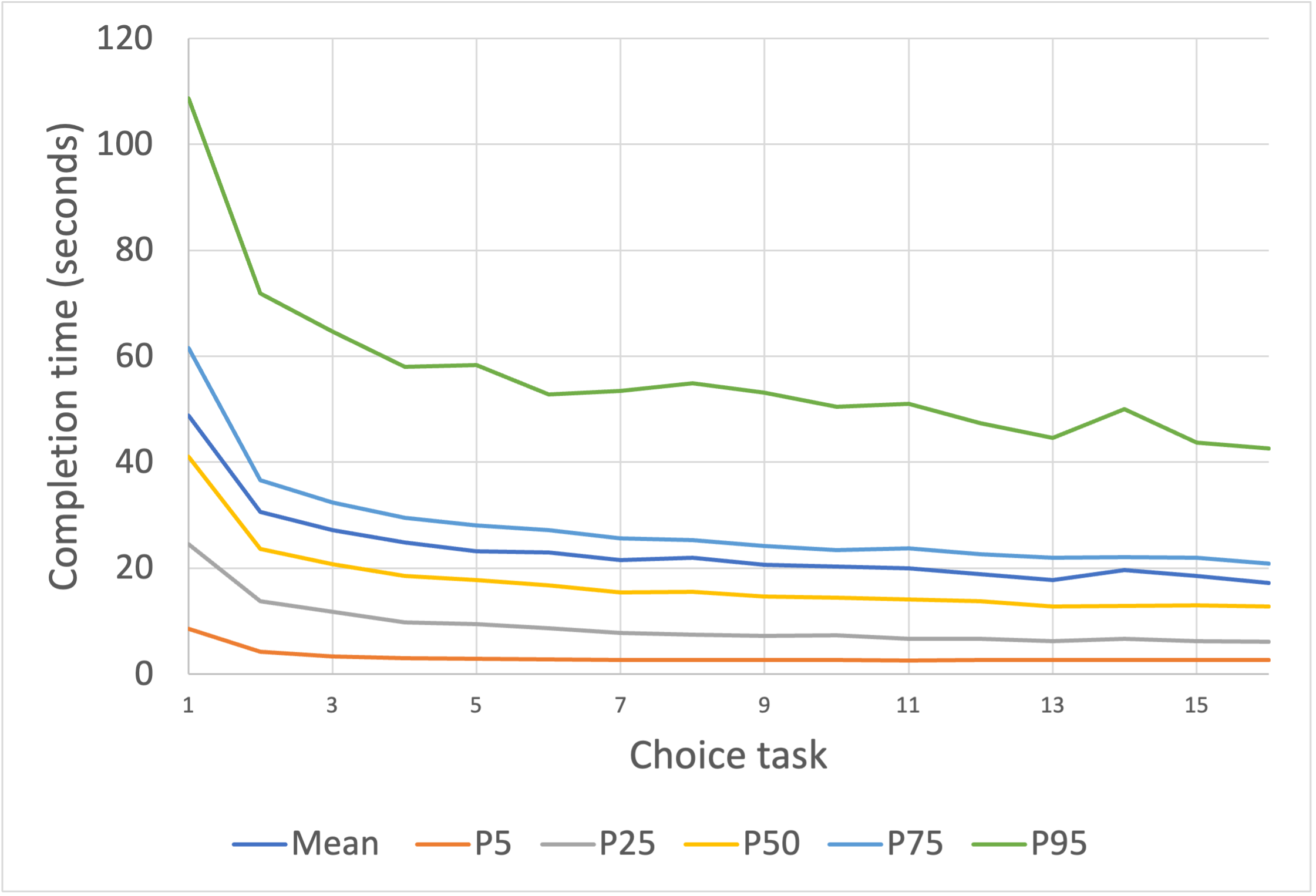
**

**Supplementary Figure D.** Relationship of completion time decile with model fit and number of statistically significant coefficients


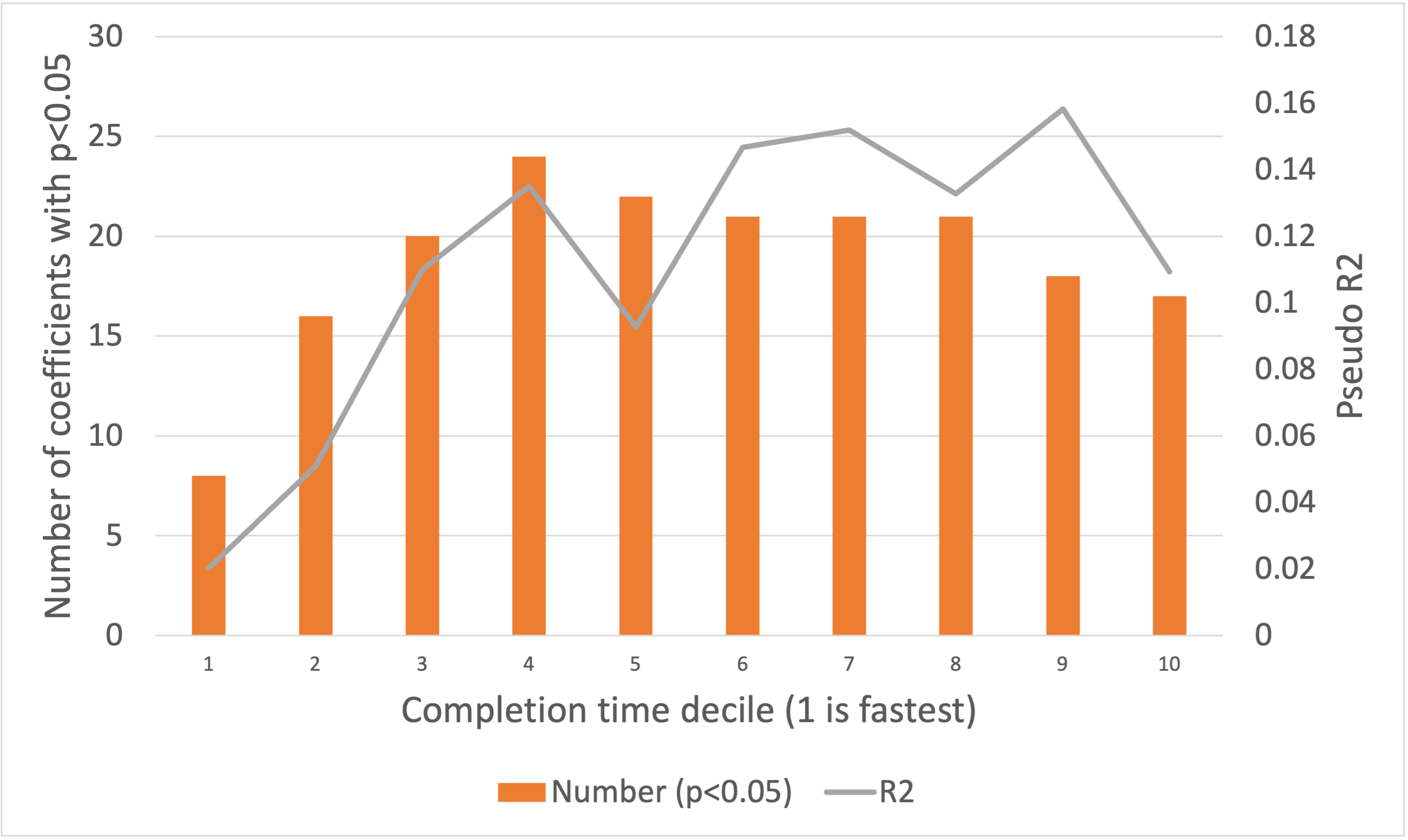

Supplement: Supplementary file 5 — Supplementary Material 5 [file 12955_2025_2442_MOESM5_ESM.docx]
