# Supplementary material for: Japanese value set for the Functional Assessment of Cancer Therapy Eight Dimension (FACT-8D) cancer-specific preference-based quality of life instrument
Source: Health Qual Life Outcomes. 2025 Oct 29;23:109. doi: 10.1186/s12955-025-02442-3 (PMC12574001; doi:10.1186/s12955-025-02442-3)
Supplement: Supplementary file 6 — Supplementary Material 6 [file 12955_2025_2442_MOESM6_ESM.docx]

**Supplementary Table B.** Conditional logit results for Model 1 (unconstrained, unweighted) and Model 2 (monotonicity imposed, unweighted and weighted) (estimated coefficients and robust standard errors (SE)), based on data from respondents who completed at least one choice pair in the discrete choice experiment (n=2445)

| Coefficient (SE)^a^ |  | Unweighted Analysis | Weighted Analysis^b^ | | Uncertainty inflation % (unconstrained)^c^ |
| --- | --- | --- | --- | --- | --- |
| Dimension | Level | Unconstrained | Unconstrained | Constrained |  |
| Duration | Linear (years) | 0.4366 (0.0144)*** | 0.4328 (0.0253)*** | 0.4362 (0.0246)*** | 75.7 |
| Duration x Pain | 2 | -0.0230 (0.0059)*** | -0.0192 (0.0083)** | -0.0193 (0.0083)** | 40.7 |
|  | 3 | -0.0335 (0.0063)*** | -0.0391 (0.0100)*** | -0.039 (0.0099)*** | 58.7 |
|  | 4 | -0.0908 (0.0066)*** | -0.0937 (0.0097)*** | -0.0937 (0.0097)*** | 47.0 |
|  | 5 | -0.1399 (0.0068)*** | -0.1406 (0.0092)*** | -0.1405 (0.0093)*** | 35.3 |
| Duration x Fatigue | 2 | -0.0034 (0.0059) | 0.0052 (0.0098) | 0 | 66.1 |
|  | 3 | -0.0045 (0.0061) | -0.0085 (0.0097) | -0.0112 (0.0074) | 59.0 |
|  | 4 | -0.0453 (0.0060)*** | -0.0453 (0.0100)*** | -0.0479 (0.0078)*** | 66.7 |
|  | 5 | -0.0510 (0.0058)*** | -0.0498 (0.0092)*** | -0.0523 (0.0074)*** | 58.6 |
| Duration x Nausea | 2 | -0.0309 (0.0047)*** | -0.0275 (0.0067)*** | -0.0274 (0.0067)*** | 42.6 |
|  | 3 | -0.0481 (0.0052)*** | -0.0373 (0.0091)*** | -0.037 (0.0092)*** | 75.0 |
|  | 4 | -0.0829 (0.0056)*** | -0.0755 (0.0090)*** | -0.0751 (0.0091)*** | 60.7 |
|  | 5 | -0.1291 (0.0054)*** | -0.1279 (0.0085)*** | -0.1277 (0.0086)*** | 57.4 |
| Duration x Sleep | 2 | -0.0179 (0.0062)*** | -0.0123 (0.0098) | -0.0125 (0.0097) | 58.1 |
|  | 3 | -0.0229 (0.0056)*** | -0.0146 (0.0100) | -0.0145 (0.0101) | 78.6 |
|  | 4 | -0.0530 (0.0058)*** | -0.0457 (0.0097)*** | -0.0457 (0.0097)*** | 67.2 |
|  | 5 | -0.0821 (0.0060)*** | -0.0706 (0.0118)*** | -0.0707 (0.0118)*** | 96.7 |
| Duration x Work | 2 | -0.0153 (0.0048)*** | -0.0177 (0.0088)** | -0.0177 (0.0088)** | 83.3 |
|  | 3 | -0.0351 (0.0049)*** | -0.0368 (0.0084)*** | -0.0367 (0.0084)*** | 71.4 |
|  | 4 | -0.0738 (0.0051)*** | -0.0832 (0.0080)*** | -0.0831 (0.008)*** | 56.9 |
|  | 5 | -0.1024 (0.0053)*** | -0.1155 (0.0082)*** | -0.1154 (0.0082)*** | 54.7 |
| Duration x Support | 2 | -0.0023 (0.0055) | -0.0077 (0.0080) | -0.0063 (0.0074) | 45.5 |
|  | 3 | -0.0139 (0.0059)** | -0.0047 (0.0095) | -0.0063 (0.0074) | 61.0 |
|  | 4 | -0.0488 (0.0060)*** | -0.0535 (0.0105)*** | -0.0538 (0.0105)*** | 75.0 |
|  | 5 | -0.0635 (0.0057)*** | -0.0591 (0.0100)*** | -0.0594 (0.0099)*** | 75.4 |
| Duration x Sadness | 2 | -0.0202 (0.0060)*** | -0.0140 (0.0095) | -0.0142 (0.0094) | 58.3 |
|  | 3 | -0.0219 (0.0057)*** | -0.0240 (0.0085)*** | -0.0243 (0.0085)*** | 49.1 |
|  | 4 | -0.0547 (0.0058)*** | -0.0598 (0.0095)*** | -0.0599 (0.0096)*** | 63.8 |
|  | 5 | -0.0742 (0.0061)*** | -0.0763 (0.0097)*** | -0.0764 (0.0096)*** | 59.0 |
| Duration x Worry | 2 | -0.0006 (0.0060) | 0.0012 (0.0123) | 0 | 105.0 |
|  | 3 | -0.0111 (0.0055)** | -0.0167 (0.0092)* | -0.0173 (0.0078)** | 67.3 |
|  | 4 | -0.0372 (0.0056)*** | -0.0446 (0.0099)*** | -0.0454 (0.0069)*** | 76.8 |
|  | 5 | -0.0543 (0.0059)*** | -0.0667 (0.0094)*** | -0.0673 (0.0079)*** | 59.3 |
| Pseudo R2 | | 0.0955 | 0.0980 | 0.0980 |  |
| Log Pseudo-likelihood | | -23733.437 | 22137.208 | 22137.796 |  |
| Akaike information criterion (AIC) | | 47533 | 44340 | 44336 |  |
| Bayesian information criterion (BIC) | | 47838 | 44645 | 44613 |  |

a. The coefficient for each level of each QOL domain was estimated as the interaction of that level with duration. Levels combined to ensure monotonicity within each dimension are noted in italics. Statistical significance: ***0.1%; **1%; *5%.

b. Analyses were weighted for five variables simultaneously using raking: education, work type, household income, health status (EQ-5D), mental health (Kessler 6).

c. Variance inflation expressed as percentage increase in Model 1 coefficient SE = (weighted SE − unweighted SE)/unweighted SE; average (Av.), minimum (Min.), median (Med.), maximum (Max.).
