# Supplementary material for: Japanese value set for the Functional Assessment of Cancer Therapy Eight Dimension (FACT-8D) cancer-specific preference-based quality of life instrument
Source: Health Qual Life Outcomes. 2025 Oct 29;23:109. doi: 10.1186/s12955-025-02442-3 (PMC12574001; doi:10.1186/s12955-025-02442-3)
Supplement: Supplementary file 7 — Supplementary Material 7 [file 12955_2025_2442_MOESM7_ESM.docx]

**Online resource 7**

**Supplementary Table C.** Latent class model results for two, three, four and five classes

| Coefficient (SE)^a^ |  | 2 classes | 3 classes | 4 classes | 5 classes |
| --- | --- | --- | --- | --- | --- |
| Dimension | Level | Unconstrained | Unconstrained | Unconstrained | Unconstrained |
| Duration | Linear (years) | 0.6838 | 1.1848 | 1.2657 | 1.4036 |
| Duration x Pain | 2 | -0.0214 | -0.0406 | -0.0671 | -0.0474 |
|  | 3 | -0.0559 | -0.0839 | -0.1047 | -0.0996 |
|  | 4 | -0.1431 | -0.1967 | -0.2513 | -0.2561 |
|  | 5 | -0.2030 | -0.2783 | -0.3167 | -0.3588 |
| Duration x Fatigue | 2 | -0.0127 | -0.0135 | -0.0193 | -0.0019 |
|  | 3 | -0.0141 | -0.0219 | -0.0332 | -0.0136 |
|  | 4 | -0.0585 | -0.0714 | -0.0983 | -0.0639 |
|  | 5 | -0.0713 | -0.0862 | -0.1245 | -0.1061 |
| Duration x Nausea | 2 | -0.0296 | -0.0486 | -0.0551 | -0.0679 |
|  | 3 | -0.0551 | -0.0936 | -0.1070 | -0.1316 |
|  | 4 | -0.1056 | -0.1589 | -0.1694 | -0.2063 |
|  | 5 | -0.1641 | -0.2305 | -0.2764 | -0.3016 |
| Duration x Sleep | 2 | -0.0353 | -0.0367 | -0.0574 | -0.0433 |
|  | 3 | -0.0423 | -0.0481 | -0.0659 | -0.0643 |
|  | 4 | -0.0768 | -0.0809 | -0.1056 | -0.1206 |
|  | 5 | -0.1171 | -0.1218 | -0.1539 | -0.1719 |
| Duration x Work | 2 | -0.0152 | -0.0300 | -0.0252 | -0.0394 |
|  | 3 | -0.0447 | -0.0775 | -0.0696 | -0.0933 |
|  | 4 | -0.0973 | -0.1433 | -0.1475 | -0.1779 |
|  | 5 | -0.1361 | -0.2040 | -0.2362 | -0.2628 |
| Duration x Support | 2 | -0.0001 | -0.0003 | 0.0000 | 0.0087 |
|  | 3 | -0.0090 | -0.0037 | -0.0502 | -0.0076 |
|  | 4 | -0.0504 | -0.0514 | -0.0900 | -0.0814 |
|  | 5 | -0.0814 | -0.0949 | -0.1415 | -0.1423 |
| Duration x Sadness | 2 | -0.0283 | -0.0319 | -0.0285 | -0.0557 |
|  | 3 | -0.0335 | -0.0637 | -0.0520 | -0.0636 |
|  | 4 | -0.0721 | -0.1217 | -0.1081 | -0.1236 |
|  | 5 | -0.0993 | -0.1601 | -0.1600 | -0.1955 |
| Duration x Worry | 2 | -0.0072 | 0.0188 | -0.0029 | -0.0198 |
|  | 3 | -0.0184 | -0.0392 | -0.0261 | -0.0489 |
|  | 4 | -0.0536 | -0.0394 | -0.0806 | -0.0928 |
|  | 5 | -0.0741 | -0.1192 | -0.1190 | -0.1251 |
| Akaike information criterion (AIC) | | 44435 | 43959 | 43503 | 44039 |
| Bayesian information criterion (BIC) | | 45053 | 44890 | 44747 | 45599 |
