# Supplementary material for: Japanese value set for the Functional Assessment of Cancer Therapy Eight Dimension (FACT-8D) cancer-specific preference-based quality of life instrument
Source: Health Qual Life Outcomes. 2025 Oct 29;23:109. doi: 10.1186/s12955-025-02442-3 (PMC12574001; doi:10.1186/s12955-025-02442-3)
Supplement: Supplementary file 8 — Supplementary Material 8 [file 12955_2025_2442_MOESM8_ESM.docx]

**Online resource 8**

**Supplementary Figure E.** Utility decrements estimated by the unweighted Model 2 (i.e. constrained) conditional logit model (blue) and the corresponding 4-class latent class model (orange)


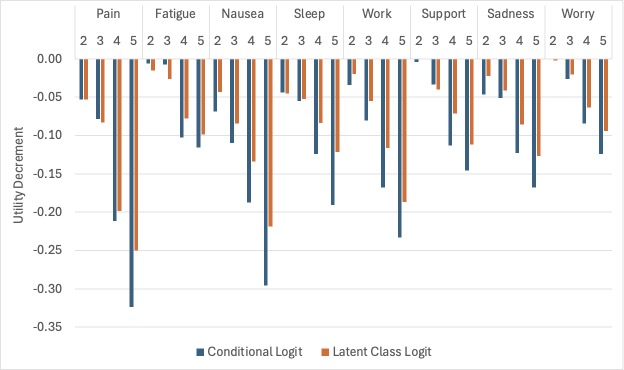


**Supplementary Table D.** Utility decrements estimated by the unweighted constrained conditional logit model and the corresponding 4-class latent class logit model

| Dimension | Level | Conditional Logit | Latent Class Logit |
| --- | --- | --- | --- |
| Pain | 2 | -0.0532 | -0.0530 |
|  | 3 | -0.0783 | -0.0827 |
|  | 4 | -0.2118 | -0.1985 |
|  | 5 | -0.3234 | -0.2502 |
| Fatigue | 2 | -0.0062 | -0.0152 |
|  | 3 | -0.0076 | -0.0262 |
|  | 4 | -0.1024 | -0.0777 |
|  | 5 | -0.1156 | -0.0984 |
| Nausea | 2 | -0.0684 | -0.0435 |
|  | 3 | -0.1100 | -0.0845 |
|  | 4 | -0.1876 | -0.1338 |
|  | 5 | -0.2953 | -0.2184 |
| Sleep | 2 | -0.0437 | -0.0454 |
|  | 3 | -0.0548 | -0.0521 |
|  | 4 | -0.1243 | -0.0834 |
|  | 5 | -0.1908 | -0.1216 |
| Work | 2 | -0.0338 | -0.0199 |
|  | 3 | -0.0803 | -0.0550 |
|  | 4 | -0.1680 | -0.1165 |
|  | 5 | -0.2332 | -0.1866 |
| Support | 2 | -0.0039 | 0.0000 |
|  | 3 | -0.0336 | -0.0397 |
|  | 4 | -0.1130 | -0.0711 |
|  | 5 | -0.1455 | -0.1118 |
| Sadness | 2 | -0.0463 | -0.0225 |
|  | 3 | -0.0509 | -0.0411 |
|  | 4 | -0.1227 | -0.0854 |
|  | 5 | -0.1680 | -0.1264 |
| Worry | 2 | 0.0000 | -0.0023 |
|  | 3 | -0.0260 | -0.0206 |
|  | 4 | -0.0843 | -0.0637 |
|  | 5 | -0.1238 | -0.0940 |
