# Supplementary material for: Japanese value set for the Functional Assessment of Cancer Therapy Eight Dimension (FACT-8D) cancer-specific preference-based quality of life instrument
Source: Health Qual Life Outcomes. 2025 Oct 29;23:109. doi: 10.1186/s12955-025-02442-3 (PMC12574001; doi:10.1186/s12955-025-02442-3)
Supplement: Supplementary file 9 — Supplementary Material 9 [file 12955_2025_2442_MOESM9_ESM.docx]

**Online resource 9**

**Supplementary Appendix D. Instructions for calculating FACT-8D scores from FACT-G responses using Japanese general population preference weights, with STATA and SPSS code**

To calculate the FACT-8D score from FACT-G responses of a particular patient *p*, first determine their FACT-8D level *l* for each dimension *d*, following the mapping of FACT-G items to FACT-8D levels in Online Resource 1.

A utility score of 1 is assigned to patients whose FACT-G scores indicate they are at level 1 of all 10 dimensions of the FACT-8D. For all other health states, the utility score is 1 minus each the utility decrement (*wdl*) for each level down from no problems in each of the 8 FACT-8D dimensions.

**STATA code to calculate FACT-8D utility scores from FACT-G responses using Japanese utility set**

* Written and tested by Richard Norman richard.norman@curtin.edu.au July 2024

* This code is designed to convert FACT-G responses into FACT-8D utility weights.

* It uses the Japanese DCE-derived weights developed by Shiroiwa, King, Norman et al.

* It is based on the assumption that the underlying data are coded between 0 and 4 where 0

* means 'Not at all', 1 means 'A little bit', 2 means 'Somewhat', 3 means 'Quite a bit', and 4

* means 'Very much'. The coding of the variables is clustered by domain, so Physical Well-Being

* items are labelled GP1-GP7, Social / Family Well-Being items are labelled GS1-GS7,

* Emotional Well-Being items are labelled GE1-GE6, and Functional Well-Being are labelled

* GF1-GF7.

gen pai = gp4

gen fat = gp1

gen nau = gp2

gen sle = 4-gf5

gen wrk = 4-gf1

gen sup = 4 - max(gs2,gs3)

gen sad = ge1

gen wor = ge6

gen paidec=.

replace paidec=0 if pai==0

replace paidec=-0.0531 if pai==1

replace paidec=-0.0782 if pai==2

replace paidec=-0.2117 if pai==3

replace paidec=-0.3234 if pai==4

gen fatdec=.

replace fatdec=0 if fat==0

replace fatdec=-0.0062 if fat==1

replace fatdec=-0.0076 if fat==2

replace fatdec=-0.1024 if fat==3

replace fatdec=-0.1155 if fat==4

gen naudec=.

replace naudec=0 if nau==0

replace naudec=-0.0683 if nau==1

replace naudec=-0.1100 if nau==2

replace naudec=-0.1877 if nau==3

replace naudec=-0.2953 if nau==4

gen sledec=.

replace sledec=0 if sle==0

replace sledec=-0.0437 if sle==1

replace sledec=-0.0547 if sle==2

replace sledec=-0.1244 if sle==3

replace sledec=-0.1907 if sle==4

gen wrkdec=.

replace wrkdec=0 if wrk==0

replace wrkdec=-0.0337 if wrk==1

replace wrkdec=-0.0803 if wrk==2

replace wrkdec=-0.1680 if wrk==3

replace wrkdec=-0.2332 if wrk==4

gen supdec=.

replace supdec=0 if sup==0

replace supdec=-0.0040 if sup==1

replace supdec=-0.0336 if sup==2

replace supdec=-0.1131 if sup==3

replace supdec=-0.1456 if sup==4

gen saddec=.

replace saddec=0 if sad==0

replace saddec=-0.0462 if sad==1

replace saddec=-0.0508 if sad==2

replace saddec=-0.1227 if sad==3

replace saddec=-0.1681 if sad==4

gen wordec=.

replace wordec=0 if wor==0

replace wordec=0 if wor==1

replace wordec=-0.0261 if wor==2

replace wordec=-0.0842 if wor==3

replace wordec=-0.1239 if wor==4

gen fact8d = 1 + paidec + fatdec + naudec + sledec + wrkdec + supdec + saddec + wordec

**SPSS syntax SPSS code to calculate FACT-8D utility scores from FACT-G responses using Japanese utility set**

Written by Daniel Costa daniel.costa@sydney.edu.au 16th December 2020

Adapted and tested in 2024 for the Japanese value set by Rachel Campbell

* Encoding: UTF-8.

* Encoding: .

* This code is designed to convert FACT-G responses into FACT-8D utility weights.

* It uses the Japanese DCE-derived weights developed by Shiroiwa, King, Norman et al.

* It is based on the assumption that the underlying data are coded between 0 and 4

* where 0 means 'Not at all', 1 means 'A little bit', 2 means 'Somewhat',

* 3 means 'Quite a bit', and 4 means 'Very much'. The coding of the variables

* is clustered by domain, so Physical Well-Being items are labelled GP1-GP7,

* Social / Family Well-Being items are labelled GS1-GS7, Emotional Well-Being items

* are labelled GE1-GE6, and Functional Well-Being are labelled GF1-GF7.

compute pai = gp4.

compute fat = gp1.

compute nau = gp2.

compute sle = 4-gf5.

compute wrk = 4-gf1.

compute sup = 4 - max(gs2,gs3).

compute sad = ge1.

compute wor = ge6.

exe.

compute paidec=$sysmis.

if pai=0 paidec=0.

if pai=1 paidec=-0.0531.

if pai=2 paidec=-0.0782.

if pai=3 paidec=-0.2117.

if pai=4 paidec=-0.3234.

compute fatdec= $sysmis.

if fat=0 fatdec=0.

if fat=1 fatdec=-0.0062.

if fat=2 fatdec=-0.0076.

if fat=3 fatdec=-0.1024.

if fat=4 fatdec=-0.1155.

compute naudec=$sysmis.

if nau=0 naudec=0.

if nau=1 naudec=-0.0683.

if nau=2 naudec=-0.1100.

if nau=3 naudec=-0.1877.
if nau=4 naudec=-0.2953.

compute sledec=$sysmis.

if sle=0 sledec=0.

if sle=1 sledec=-0.0437.

if sle=2 sledec=-0.0547.

if sle=3 sledec=-0.1244.

if sle=4 sledec=-0.1907.

compute wrkdec=$sysmis.

if wrk=0 wrkdec=0.

if wrk=1 wrkdec=-0.0337.

if wrk=2 wrkdec=-0.0803.

if wrk=3 wrkdec=-0.1680.

if wrk=4 wrkdec=-0.2332.

compute supdec=$sysmis.

if sup=0 supdec=0.

if sup=1 supdec=-0.0040.

if sup=2 supdec=-0.0336.

if sup=3 supdec=-0.1131.

if sup=4 supdec=-0.1456.

compute saddec=$sysmis.

if sad=0 saddec=0.

if sad=1 saddec=-0.0462.

if sad=2 saddec=-0.0508.

if sad=3 saddec=-0.1227.

if sad=4 saddec=-0.1681.

compute wordec=$sysmis.

if wor=0 wordec=0.

if wor=1 wordec=0.

if wor=2 wordec=-0.0261.

if wor=3 wordec=-0.0842.

if wor=4 wordec=-0.1239.

compute fact8d = 1 + paidec + fatdec + naudec + sledec + wrkdec + supdec + saddec + wordec. exe.
